# Discovery of the novel autophagy inhibitor aumitin that targets mitochondrial complex I†

**DOI:** 10.1039/c7sc05040b

**Published:** 2018-02-26

**Authors:** Lucas Robke, Yushi Futamura, Georgios Konstantinidis, Julian Wilke, Harumi Aono, Zhwan Mahmoud, Nobumoto Watanabe, Yao-Wen Wu, Hiroyuki Osada, Luca Laraia, Herbert Waldmann

**Affiliations:** a Max-Planck-Institute of Molecular Physiology, Department of Chemical Biology Otto-Hahn-Str. 11 44227 Dortmund Germany; b Faculty of Chemistry and Chemical Biology, TU Dortmund University Otto-Hahn-Str. 4a 44227 Dortmund Germany herbert.waldmann@mpi-dortmund.mpg.de; c RIKEN-Max Planck Joint Research Division for Systems Chemical Biology, RIKEN CSRS 2-1, Hirosawa, Wako Saitama 351-0198 Japan; d Chemical Biology Research Group, RIKEN CSRS 2-1, Hirosawa, Wako Saitama 351-0198 Japan; e Chemical Genomics Centre of the Max-Planck-Society Otto-Hahn-Str. 15 44227 Dortmund Germany; f Bio-Active Compounds Discovery Research Unit, RIKEN CSRS 2-1, Hirosawa, Wako Saitama 351-0198 Japan

## Abstract

Macroautophagy is a conserved eukaryotic process for degradation of cellular components in response to lack of nutrients. It is involved in the development of diseases, notably cancer and neurological disorders including Parkinson's disease. Small molecule autophagy modulators have proven to be valuable tools to dissect and interrogate this crucial metabolic pathway and are in high demand. Phenotypic screening for autophagy inhibitors led to the discovery of the novel autophagy inhibitor aumitin. Target identification and confirmation revealed that aumitin inhibits mitochondrial respiration by targeting complex I. We show that inhibition of autophagy by impairment of mitochondrial respiration is general for several mitochondrial inhibitors that target different mitochondrial complexes. Our findings highlight the importance of mitochondrial respiration for autophagy regulation.

## Abstract 2

Phenotypic screening for autophagy inhibitors followed by target identification resulted in the discovery of aumitin, a new autophagy inhibitor that inhibits mitochondrial respiration by targeting complex I.

## Graphical abstract

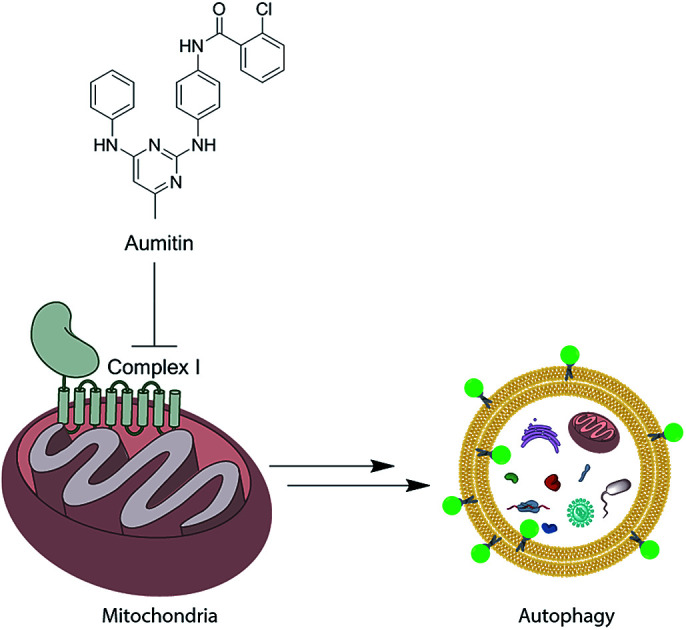

Macroautophagy (henceforth autophagy) is a conserved, eukaryotic process essential for cellular development, homeostasis and survival.^1,2^ It plays a role in the prevention of cancer by removing damaged organelles including dysfunctional mitochondria, in the cell.^3^ It also promotes the survival of cancer cells under stress conditions including nutrient deprivation.^1^ During autophagy, cellular components are sequestered, engulfed by the phagophore, the precursor to the autophagosome, and subsequently eliminated through autophagosome–lysosome fusion.^4^ Autophagy is tightly connected to metabolism,^5^ nutrient uptake and cellular energy supply by the mitochondria.^6^ Mitochondria regulate autophagy by generation of ATP and production of reactive oxygen species (ROS).^7^ Conversely autophagy controls mitochondrial homeostasis by means of mitophagy.^8^ Inhibition of autophagy has been linked to the onset of Parkinson's disease due to impaired mitochondrial turnover.^9^ Due to this interplay, small molecules that modulate autophagy through modulation of mitochondrial function are invaluable tools for the study of the biological processes involved^10–15^ and may inspire new drug discovery programs.^16^ Here we describe the discovery of aumitin, a novel diaminopyrimidine-based autophagy inhibitor which targets mitochondrial complex I. More generally we show that inhibition of mitochondrial respiration, irrespective of the targeted complex, inhibits autophagy.

To identify novel autophagy inhibitors, we employed a high-content screening approach using MCF-7 cells stably expressing the autophagosome marker, eGFP-LC3 (MCF7-LC3).^17^ Diaminopyrimidine based compounds were identified as very potent autophagy inhibitors, as exemplified by the most potent hit (1), which we termed aumitin (Fig. 1). Aumitin and analogues thereof inhibited starvation- and rapamycin induced autophagy dose dependently (Fig. 1A–D), which suggests that they might target the pathway downstream of mammalian target of rapamycin (mTOR).

**Fig. 1:**
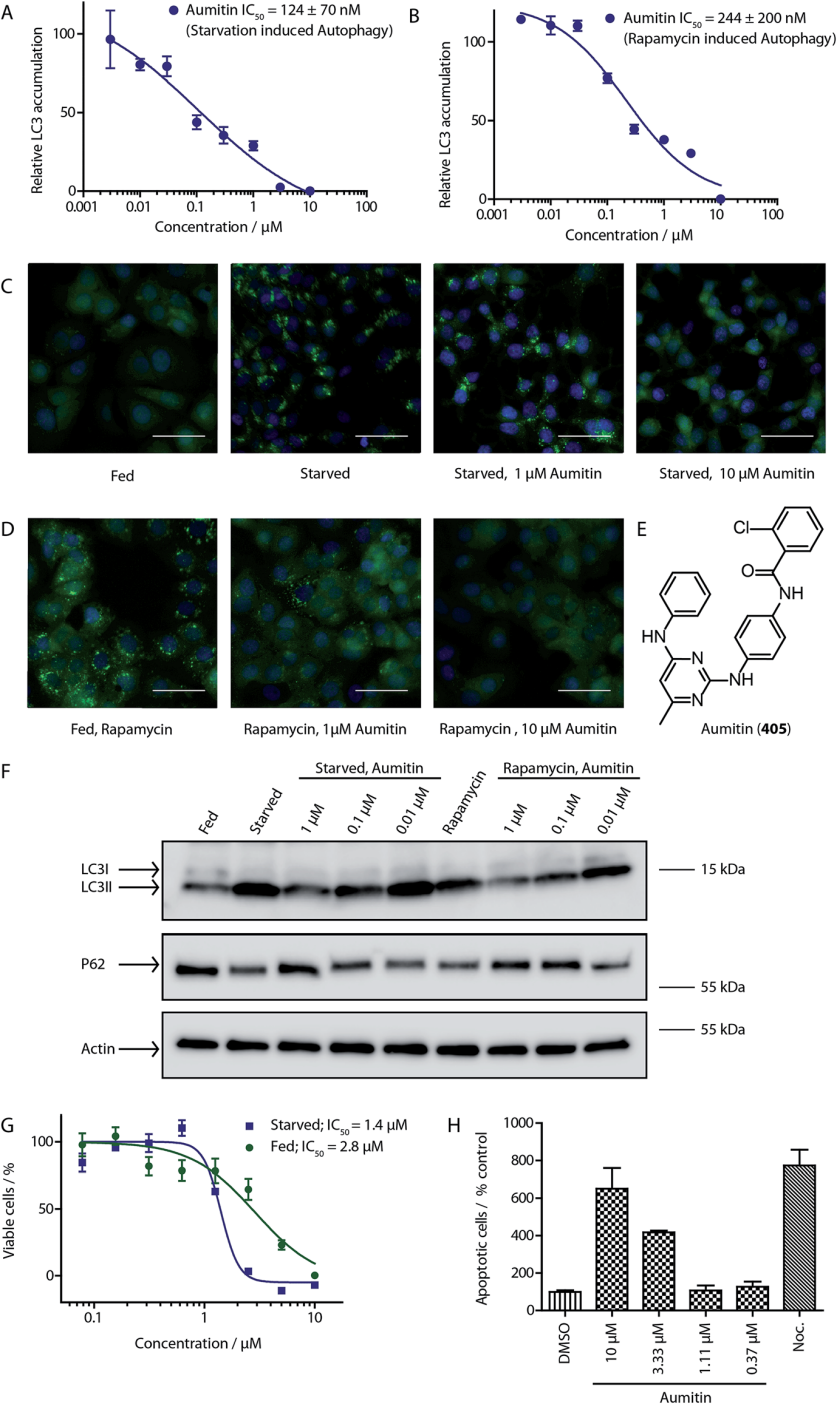
Phenotypic validation of aumitin as an autophagy inhibitor. (A–D) Phenotypic screen for inhibition of LC3 accumulation. (A) Dose-dependent inhibition of amino acid starvation induced eGFP-LC3 accumulation by aumitin. (B) Dose dependent inhibition of rapamycin induced eGFP-LC3 accumulation by aumitin. Data is mean ± SD, *n* ≥ 3, representative graphs shown. (C) Fluorescence microscopy images of the starvation induced autophagy screen. Fed = DMSO control in MEM. Starved = autophagy induced by amino acid withdrawal (EBSS). (D) Fluorescence microscopy images of the rapamycin induced autophagy screen. Rapamycin was used in MEM at 100 nM. Aumitin reverts the phenotype in a dose dependent manner. Scale bar = 50 μm. (E) Structure of aumitin. (F) Inhibition of autophagy-induced LC3-II lipidation and p62 degradation by aumitin in MCF7-LC3 cells. *n* ≥ 3, representative blot shown. (G and H) Aumitin induces cell death in starved cells by means of apoptosis. (G) Treatment of MCF7-LC3 cells under starved conditions (EBSS) or fed conditions (MEM) with aumitin. Under starvation conditions survival is reduced. Cytotoxicity was assessed by means of a WST-1 assay. Data points are mean ± SD, *n* ≥ 3, representative graph shown. (H) Aumitin dose dependently induces apoptosis in starved MCF7 cells, as assessed by using a selective caspase 3/7 probe in an IncuCyte Zoom live-cell microscope. Noc. = nocodazole (10 μM), data points are mean ± SD, *n* ≥ 3, representative experiment shown.

Aumitin (for a synthesis see ESI Fig. 1), was chosen for in depth-characterization, as it was the most potent diaminopyrimidine inhibitor. Upon autophagy induction, the cytosolic protein microtubule-associated protein light chain 3 (LC3-I) is conjugated to phosphatidylethanolamine (PE) to become the membrane-bound form LC3-II. Aumitin inhibited LC3 lipidation in a dose-dependent manner in starved and rapamycin treated MCF7-LC3 cells (Fig. 1F and ESI Fig. 2). To examine the impact of aumitin on autophagic flux, the levels of the autophagy substrate p62 were investigated.^18^ p62 targets proteins for degradation by the autophagic machinery, where it is degraded together with its cargo. Aumitin inhibited p62 degradation by starvation- as well as rapamycin-induced autophagy dose-dependently in MCF7-LC3 cells, suggesting inhibition of autophagic flux (Fig. 1F and ESI Fig. 2†). Moreover, inhibition of autophagic flux was confirmed by an independent cellular assay using mCherry-eGFP-LC3 cells.^19^ Because eGFP fluorescence is quenched in acidic conditions while mCherry fluorescence is stable, the autophagosome and autolysosome can be distinguished by yellow puncta (eGFP + mCherry) and red puncta (mCherry only), respectively (ESI Fig. 3†). As expected for an autophagy inhibitor, aumitin enhanced death of amino acid-starved cells as compared to fed cells (Fig. 1G).^20^ Aumitin induced cell death through apoptosis, as assessed using a caspase 3/7 probe that stains apoptotic cells (Fig. 1H).

Due to aumitin's structural similarity to known kinase inhibitors,^21^ we screened for kinases that can be inhibited by aumitin at a concentration of 1 μM. Out of 419 tested kinases only the two phosphatidylinositol kinases PI4KB and PI3KC2G were weakly inhibited (ESI Table 2†). However, highly potent and selective inhibitors of these kinases did not lead to autophagy inhibition (ESI Table 3†). This suggests that PI4KB and PI3KC2G are not responsible for aumitin's effect on autophagy.

Given the intimate interplay between autophagy and cellular metabolic demand, we investigated whether aumitin interferes with key metabolic processes including glucose uptake and oxidative phosphorylation. However, aumitin did not inhibit glucose uptake^22^ in HCT116 cells (residual activity > 75% at 30 μM, *n* = 4). Mitochondria have been shown to play a role in autophagy regulation.^7,8^ To test whether aumitin interfered with mitochondrial respiration, it was tested in a cell-based assay monitoring cellular oxygen consumption using the Seahorse XFe96 Analyzer (Fig. 2 and ESI Fig. 5†). This contains two fluorophores embedded in a polymer in close proximity to the seeded cells. Quenching of one fluorophore by oxygen allows the kinetic determination of the oxygen consumption rate (OCR), which is representative for respiration. The other fluorophore is pH sensitive and indicates the extracellular acidification rate (ECAR) which mirrors anaerobic glycolysis leading to lactate excretion.

OCR and ECAR are regulated by inhibitors of mitochondrial respiration (Fig. 2). While oxygen consumption declines, extracellular acidification increases in the presence of Aumitin (Fig. 2, time point (a)). Addition of mitochondrial complex V inhibitor oligomycin at timepoint (b) leads to decrease of OCR level (Fig. 2, black line), suggesting ATP-driven respiration. Oligomycin can also be used to determine the extent of proton leak respiration, *i.e.* the influx of protons into mitochondria independent of an ATPase, through the difference in the OCR between 0 μM and 10 μM aumitin after timepoint (b). Addition of protonophore FCCP (Fig. 2, timepoint (c)) reveals the maximal respiratory capacity, as it extinguishes the electrochemical gradient, such that respiration is fastest. In order to determine non-mitochondrial respiration, *i.e.* the lowest possible OCR level, at timepoint (d) complex I inhibitor rotenone and complex III inhibitor antimycin are added together.Whereas the recently reported VPS34 inhibitor autophinib^17^ did not modulate mitochondrial respiration (ESI Fig. 4†), aumitin potently and dose dependently inhibited this process as assessed by a reduction in OCR and an increase in ECAR in MCF7 cells (Fig. 2) and HeLa cells (ESI Fig. 5†).

**Fig. 2:**
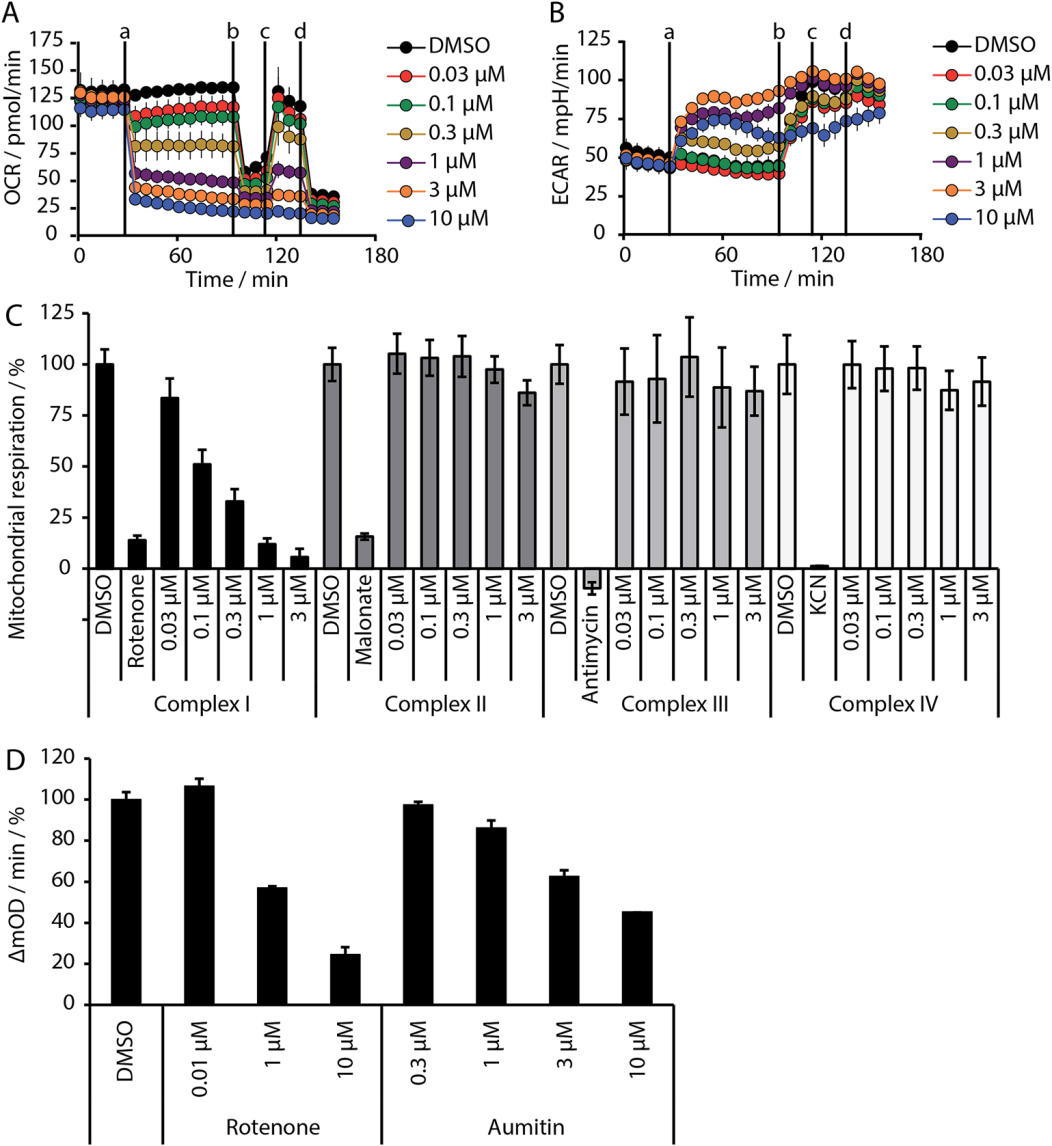
Influence of aumitin on mitochondrial respiration and identification of complex I as its target. (A and B) MCF7 cells were treated with the respective compound in a Seahorse XFe96 Analyzer, and oxygen consumption rate (OCR, (A)) and extracellular acidification rate (ECAR, (B)) were measured dose- and time-dependently. At timepoints (a) aumitin, (b) oligomycin (1 μM), (c) the decoupling agent FCCP (125 nM) and (d) rotenone and antimycin (1 μM each) were added to the samples. All data is mean ± SD, *n* = 3. (C) HeLa cells were permeabilized by digitonin and treated with the specific substrates of each mitochondrial complex. Aumitin only inhibited the pyruvate/malate-driven respiratory activity of the NADH-CoQ reductase (complex I) activity. (D) Determination of NADH-CoQ reductase activity inhibition by aumitin and rotenone in isolated mitochondria. All data is mean ± SD, *n* = 2, representative experiments shown.

Analysis of the structure activity relationship (SAR) indicated that inhibition of mitochondrial respiration in MCF7 and HeLa cells correlates strongly with inhibition of autophagy by aumitin and analogues (Table 1). Compounds with medium potency in the cell-based autophagy assay (Table 1, entries 2–5) also inhibited mitochondrial respiration less potently than aumitin. A structurally related but inactive compound in the autophagy assay did not inhibit mitochondrial respiration (Table 1, entry 6). This indicates a causal connection between the effect on mitochondrial respiration and autophagy inhibition. Due to the rapid effect of this compound class on mitochondrial respiration (Fig. 2), we speculate that the effect of mitochondrial respiration inhibition may be the cause of autophagy inhibition and not *vice versa*.

**Table 1:** Correlation of the SAR between mitochondrial respiration and autophagy. Inhibition of mitochondrial respiration was tested in a Seahorse XFe96 Analyzer by means of OCR in HeLa cells and MCF7 cells. Data is mean ± SD; *n* = 3. Inactive = no inhibition at 10 μM. Data is mean ± SD; *n* ≥ 3

Entry	Number	R^1^	R^2^	Mitochondrial respiration inhibition		Autophagy inhibition	
				HeLa cells (IC_50_ [μM])	MCF7 cells (IC_50_ [μM])	Starvation (IC_50_ [μM])	Rapamycin (IC_50_ [μM])
1	Aumitin (1)	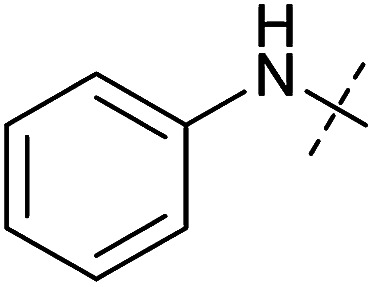	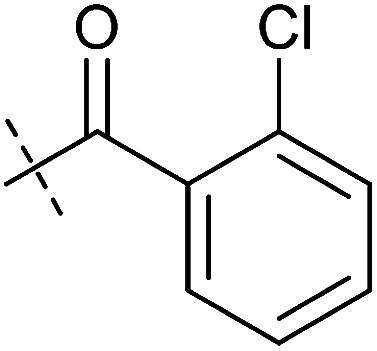	0.11 ± 0.21	0.44 ± 0.11	0.12 ± 0.07	0.24 ± 0.20
2	2	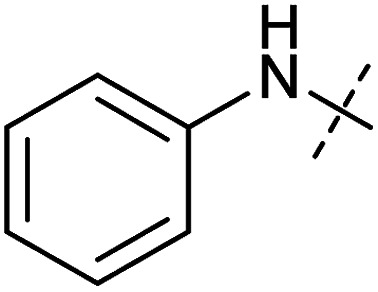	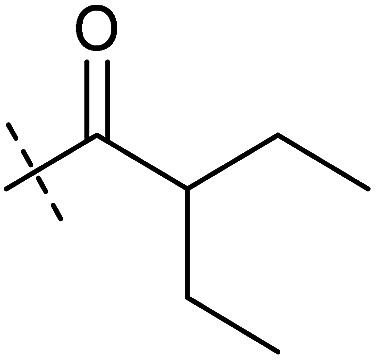	0.92 ± 0.15	1.78 ± 0.15	0.80 ± 0.30	0.81 ± 0.68
3	3	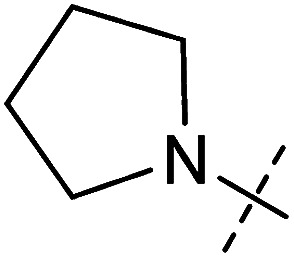	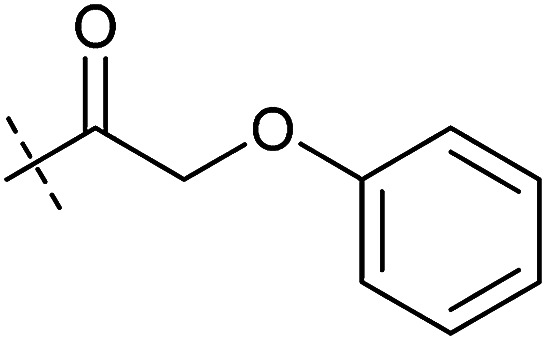	0.38 ± 0.13	0.65 ± 0.09	0.81 ± 0.16	0.29 ± 0.18
4	4	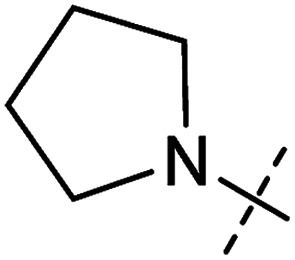	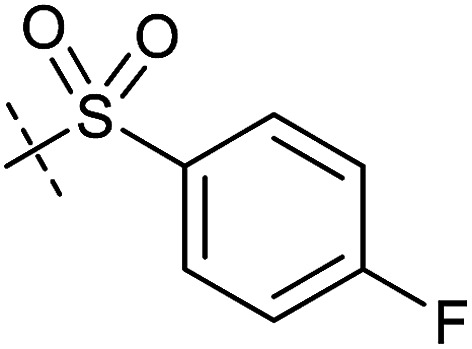	0.66 ± 0.08	1.32 ± 0.06	0.97 ± 0.47	0.16 ± 0.08
5	5	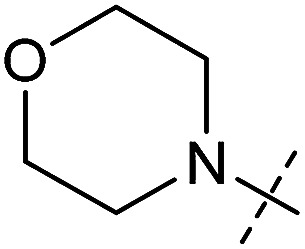	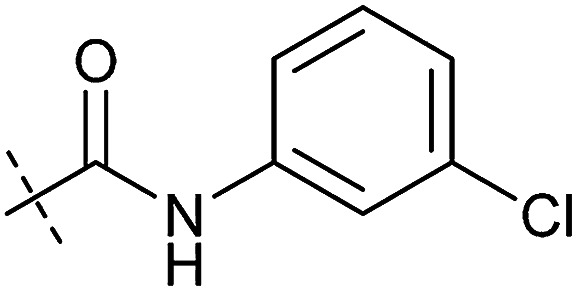	1.23 ± 0.16	8.21 ± 0.05	1.6 ± 0.60	4.1 ± 3.0
6	6	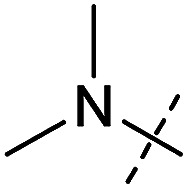	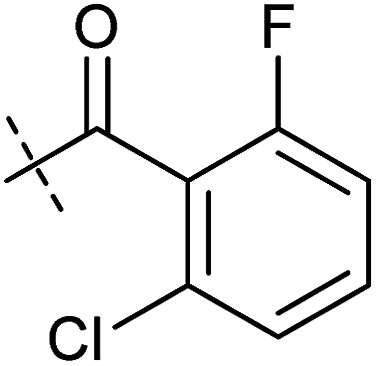	Inactive	Inactive	Inactive	Inactive


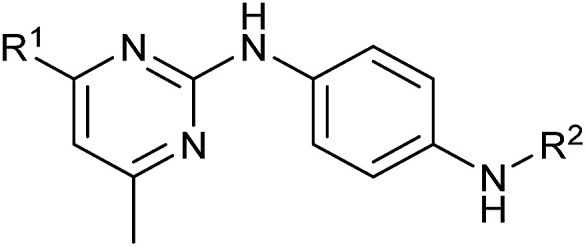
The influence of aumitin on mitochondrial respiration was investigated in greater detail by means of the previously reported semi-intact assay.^23^ The detergent digitonin is employed to permeabilize the plasma membrane, and inhibition of the different mitochondrial complexes can then be investigated by addition of their distinct substrates. In case of inhibition of a particular mitochondrial complex, only the substrates of the downstream complexes will allow continued mitochondrial respiration, as assessed by oxygen consumption. In this assay aumitin exclusively inhibited mitochondrial complex I (Fig. 2C and ESI Fig. 7†). The same result was observed for rotenone, a known inhibitor of mitochondrial complex I (ESI Fig. 6 and 7†).^13,14^

Inhibition of the pyruvate/malate-driven respiratory activity by aumitin may occur by direct binding to and inhibition of complex I, or by interception of NADH supply, which is required for the enzymatic activity of complex I. Evaluation of aumitin in an *in vitro* NADH-CoQ reductase assay using isolated mitochondria, revealed that aumitin inhibited the NADH-CoQ reductase activity, similar to rotenone (Fig. 2D). Thus, aumitin is a direct inhibitor of mitochondrial complex I.

Since aumitin and rotenone both inhibit complex I, we expected that rotenone would also potently inhibit autophagy, as it had previously been reported to do at very low concentrations (10 nM) and varying time points (2 and 24 h) in primary rat cortical neuronal cells.^13,14^ Interestingly, Giordano *et al.* showed that increased colocalisation of mitochondria with lysosomes was observed at 24 h, which is indicative of mitophagy and somewhat contradicts the fact that LC3 lipidation and p62 degradation were inhibited. However, rotenone has also been reported to decrease effective lysosomal degradation of autophagosomes,^11^ which would lead to an accumulation of autophagosomes. It has also been shown to induce autophagy in U87, HEK293 and SH-SY5Y cells; however, this has only been reported for very high concentrations (>2.5 μM) and with longer incubation times (typically >16 h).^15,24^ None-the-less, these reports may contradict our finding that aumitin inhibits autophagic flux, but also appear to show that rotenone may have different effects on autophagy depending on the time point monitored and dose used.

To clarify this discrepancy and to further confirm the link between mitochondrial respiration and autophagy, complex I inhibitors rotenone and BAY 87-2243 (ref. 25 and 26) were investigated in the eGFP-LC3 puncta assay (Table 2). Both compounds potently and dose dependently inhibited starvation- and rapamycin-induced autophagy after 3 h, similarly to aumitin. Rotenone also inhibited LC3 lipidation and p62 degradation at the same time point, which confirms its ability to inhibit autophagic flux (ESI Fig. 8†).

**Table 2:** Examination of inhibitors of mitochondrial respiration on starvation- and rapamycin-induced autophagy. Inactive = no inhibition at >10 μM. Data is mean ± SD, *n* ≥ 3

Entry	Compound name (number)	Structure	Protein target	Autophagy Inhibition	
				Starvation IC_50_ [nM]	Rapamycin IC_50_ [nM]
1	Aumitin (1)	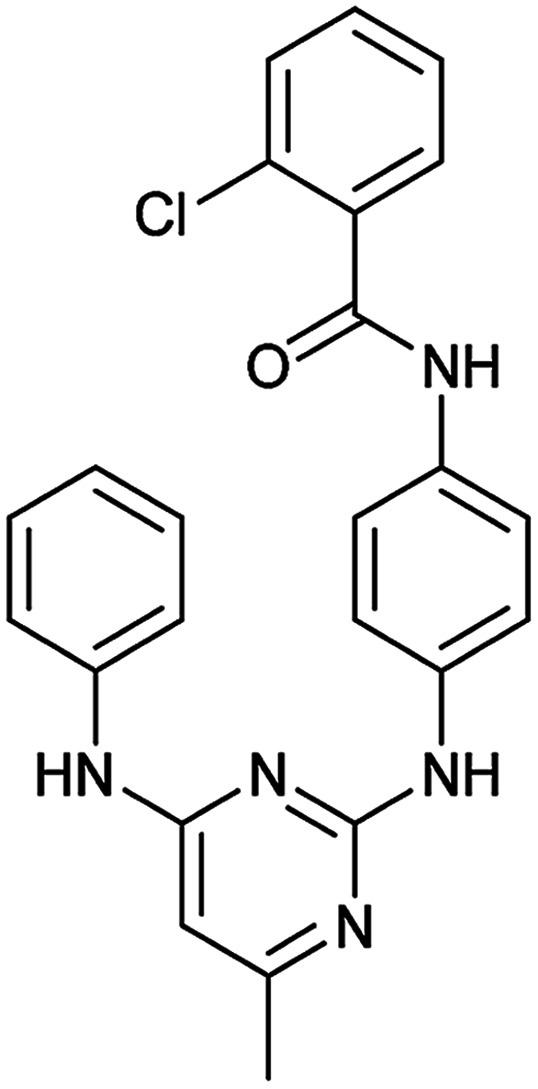	Complex I	124 ± 70	244 ± 200
2	Rotenone (7)	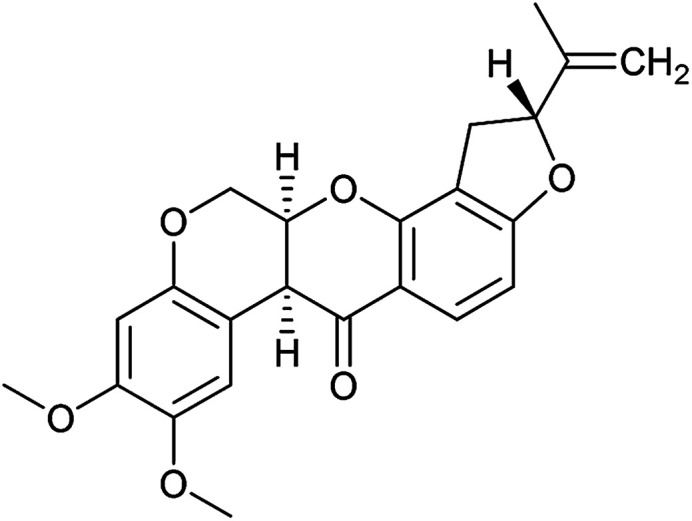	Complex I	3 ± 1	9 ± 2
3	BAY 87-2243 (8)	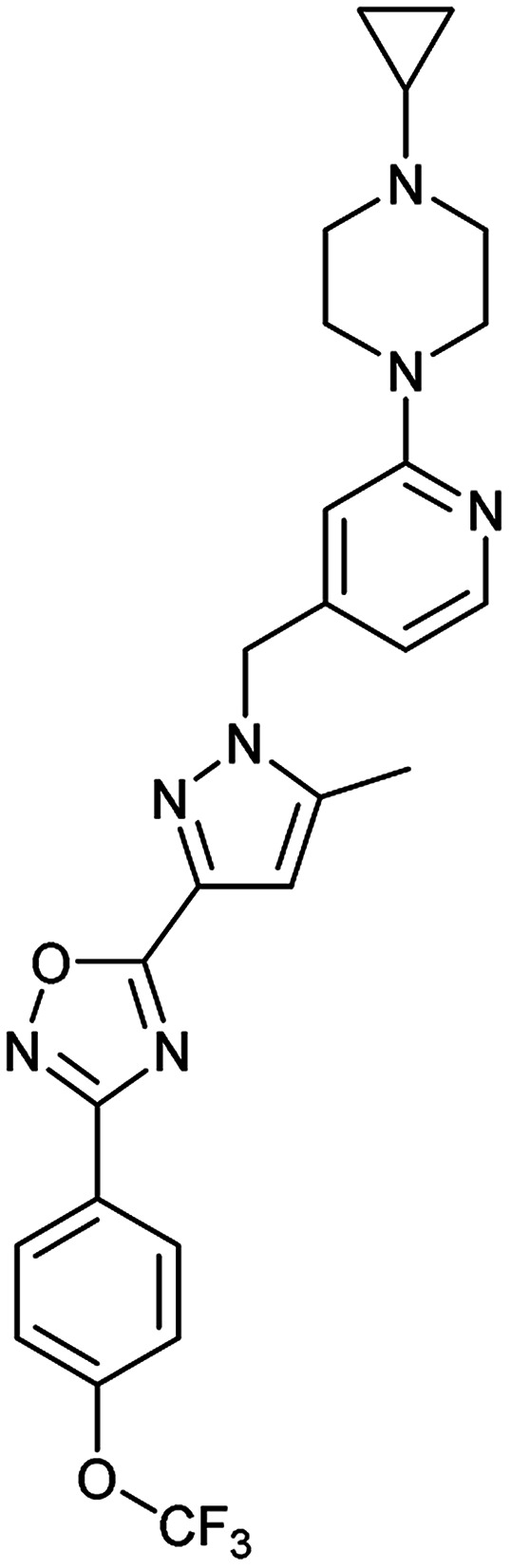	Complex I	<3	nd
4	Antimycin A (9)	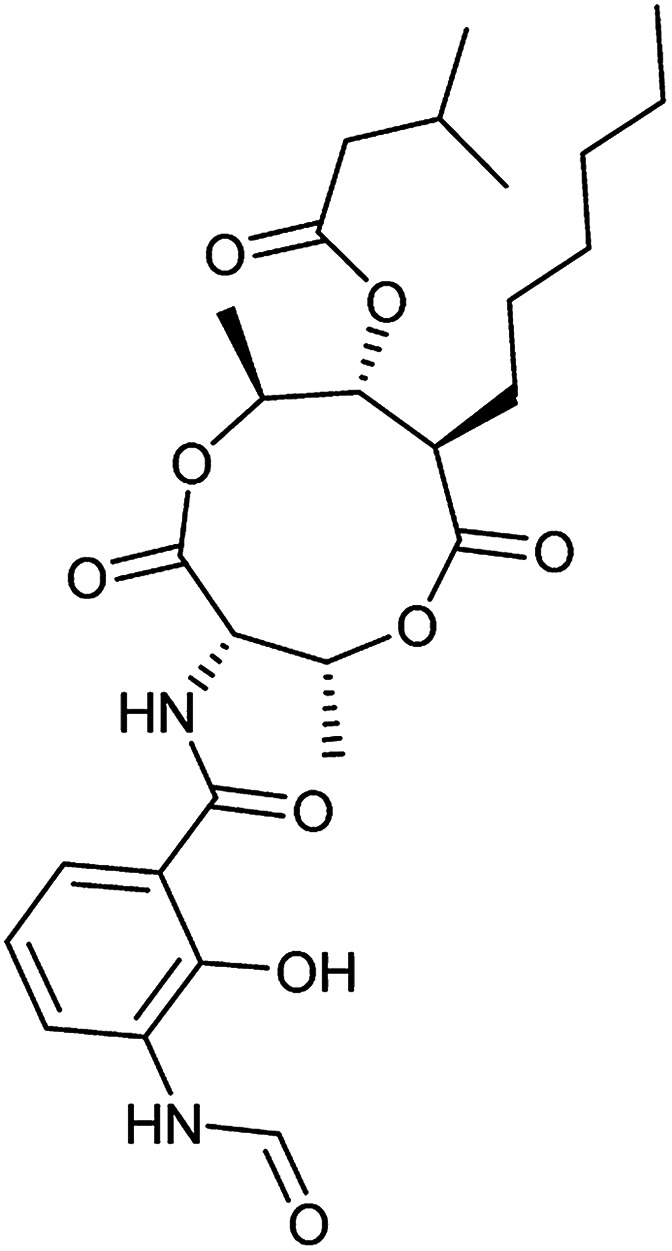	Complex III	<3	6 ± 0.7
5	Myxothiazol (10)	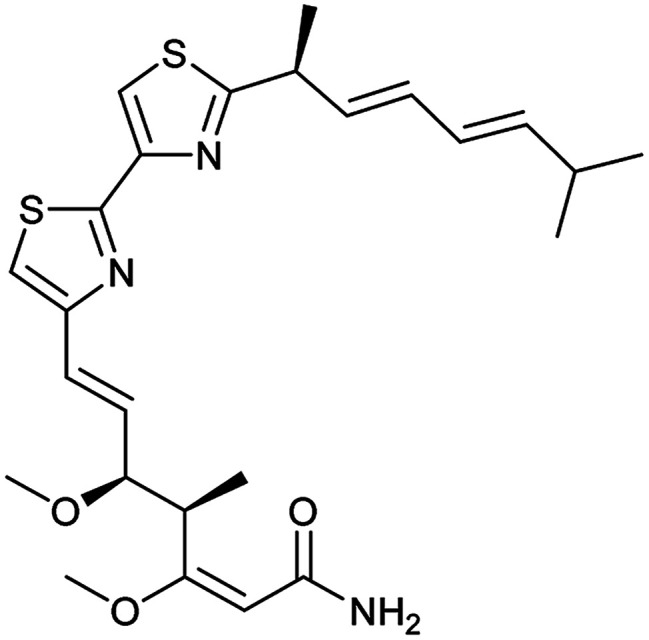	Complex III	35 ± 6	19 ± 3
6	Oligomycin A (11)	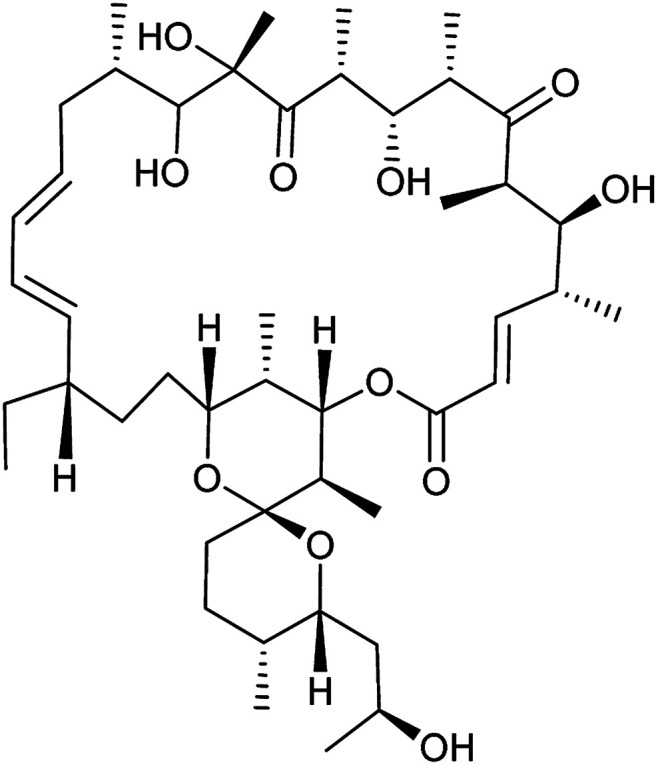	Complex V, ATP synthase	<3	7 ± 0.6
7	FCCP (12)	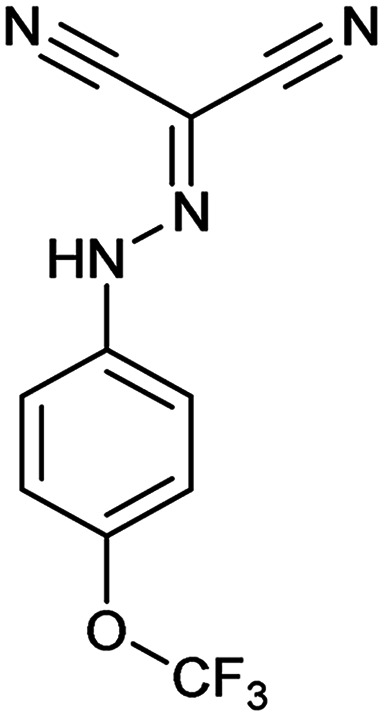	De-couples the electrochemical gradient	na (please refer to ESI Fig. 13)	643 ± 40
8	Atractyloside (13)	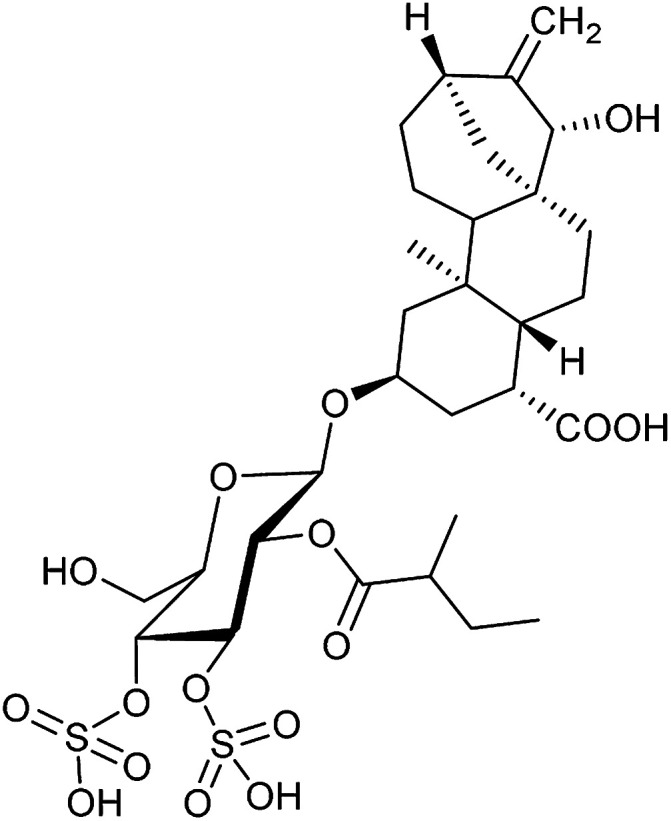	ADP/ATP translocase	Inactive	Inactive

Similarly to aumitin, rotenone inhibited the accumulation of autophagosomes and autolysosomes under fed and starved conditions (ESI Fig. 3†). These results indicate that inhibition of mitochondrial complex I downregulates autophagy prior to or at the level of autophagosome biogenesis and not maturation. Both rotenone and aumitin decreased cellular ATP levels, however at significantly higher concentrations than those required for autophagy inhibition, suggesting that ATP depletion is unlikely to be the main reason for autophagy inhibition (ESI Fig. 9†). Both compounds were also more effective at causing cell death in glucose-starved cells compared to fed cells (ESI Fig. 9 and 10†), in line with the fact that glucose depletion has been shown to sensitize cells to inhibitors of autophagy and mitochondrial respiration, significantly more than amino acid depletion (Fig. 1G).^25,26^

In order to investigate the involvement of mitochondrial respiration in autophagy we tested additional small molecules known to impair mitochondrial function. The finding that in addition to complex I inhibitors, inhibitors of complex III (Table 2, antimycin and myxothiazol, entries 4 and 5 respectively) and complex V (Table 2, oligomycin, entry 6) also potently inhibit autophagy suggests a general involvement of mitochondrial respiration in autophagy. The ADP/ATP translocase inhibitor atractyloside (Table 2, entry 8) that does not affect the mitochondrial proton gradient^27^ did not inhibit autophagy, suggesting that the latter may play a critical role in autophagy modulation, in line with previous reports.^12^ This notion was supported by the fact that the uncoupler FCCP (Table 2, entry 7) inhibits autophagy. FCCP shuttles protons across membranes from low to high pH and perturbs the formation of the proton gradient, which is necessary for proper function of ATP synthase. However, FCCP also impairs acidification of (auto)lysosomes.^28^ Thereby maturation and clearance of autophagosomal structures are impaired so that FCCP may also function as an inhibitor of autophagosome maturation. Indeed, FCCP inhibits autophagosome formation at low doses but leads to accumulation of autophagosomes at high doses as assessed by the eGFP-LC3 puncta assay and immunoblotting for LC3 lipidation (ESI Fig. 11 and 12†).

Mitochondrial respiration inhibitors have been reported to induce reactive oxygen species (ROS)^29^ which regulate autophagy by various mechanisms, including the redox inactivation of the protease ATG4 *via* oxidation at the catalytic cysteine. Thus, we investigated whether application of aumitin and rotenone increases ROS levels (ESI Fig. 13†). Aumitin and rotenone induced ROS in HeLa cells dose dependently, however only at approximately 200-fold higher concentrations than required to inhibit autophagy (ESI Fig. 13†). This finding suggests that the induction of ROS by aumitin might not be responsible for autophagy inhibition. Additionally, the known ROS inducers l-buthionine-sulfoximine (BSO) and chlorodinitrobenzene (CDNB) as well as >600 ROS inducers identified in an in-house screen (unpublished data, ESI Fig. 14 and 15†) did not inhibit autophagy, suggesting that ROS induction *per se* is unlikely to lead to autophagy inhibition. This finding is also supported by a previous report from Graef and Nunnari where overexpression of superoxide dismutase 1 or 2 (SOD) did not rescue the autophagic response in yeast treated with antimycin.^12^ They also showed that autophagy gene induction is negatively regulated by Protein Kinase A (PKA), which is activated by the inhibition of mitochondrial respiration. The exact mechanism by which mitochondrial respiration signals to PKA still remains to be elucidated.

In summary, we have discovered aumitin, a new tool compound for interrogating autophagy and mitochondrial respiration by means of complex I inhibition. We have investigated diverse inhibitors of mitochondrial respiration and shown that, irrespective of their mode of action and mitochondrial target complex, they appear to be potent autophagy inhibitors. Aumitin is a useful alternative to rotenone, and defines a novel chemotype for complex I inhibition. Rotenone has been used extensively as a chemical tool compound for the study of various pathways and for investigation of Parkinson's disease. However, rotenone also appears to have activity independent of mitochondrial complex I inhibition.^30^ Thus aumitin will be useful as a tool compound to further study the role of mitochondrial respiration and complex I in (patho)physiological processes.

## Statement of contributions

L. R. carried out the synthesis, the SAR analysis and autophagy validation experiments. Y. F. and H. A. carried out the mitochondrial respiration studies. G. K. carried out the confocal microscopy experiments. J. W. carried out the ROS experiments. Z. W. and L. L. carried out autophagy validation experiments. N. W. and H. O. supervised the mitochondrial work and analysed data. Y. W. supervised autophagy experiments. L. L. and H. W. coordinated and supervised the project. L. R., L. L. and H. W. wrote the paper, with suggestions from all co-authors.

## Conflicts of interest

There are no conflicts to declare.

## Supplementary Material

SC-009-C7SC05040B-s001
