# Supplementary material for: Discovery of the novel autophagy inhibitor aumitin that targets mitochondrial complex I
Source: Chem Sci. 2018 Feb 26;9(11):3014–22. doi: 10.1039/c7sc05040b (PMC5916016; doi:10.1039/c7sc05040b)
Supplement: SC-009-C7SC05040B-s001 [file SC-009-C7SC05040B-s001.pdf]

Electronic Supplementary Material (ESI) for Chemical Science.
This journal is © The Royal Society of Chemistry 2018

Discovery of the novel autophagy inhibitor Aumitin that targets mitochondrial complex I

Lucas Robke[a,b,c], Yushi Futamura[d], Georgios Konstantinidis[e], Julian Wilke[a,b], Harumi Aono[d], Zhwan Mahmoud[b], Nobumoto Watanabe[c,f], Yao-Wen Wu[e], Hiroyuki Osada[c,d], Luca Laraia[a,g] *, Herbert Waldmann[a,b] *

a: Max-Planck-Institute of Molecular Physiology, department of Chemical Biology, Otto-Hahn-Str. 11, 44227 Dortmund (Germany); b: Faculty of Chemistry and Chemical Biology, TU Dortmund University, Otto-Hahn-Str. 4a, 44227 Dortmund (Germany); c: RIKEN-Max Planck Joint Research Division for Systems Chemical Biology, RIKEN CSRS, 2-1, Hirosawa, Wako, Saitama 351-0198 (Japan); d: Chemical Biology Research Group, RIKEN CSRS, 2-1, Hirosawa, Wako, Saitama 351-0198 (Japan); e: Chemical Genomics Centre of the Max-Planck-Society, Otto-Hahn-Str. 15, 44227 Dortmund (Germany); f: Bio-Active Compounds Discovery Research Unit, RIKEN CSRS, 2-1, Hirosawa, Wako, Saitama 351-0198 (Japan). g: present address: Department of Chemistry, Technical University of Denmark, Kemitorvet Building 207, Room 124, 2800 Kgs. Lyngby, Denmark.

* luclar@kemi.dtu.dk, herbert.waldmann@mpi-dortmund.mpg.de

SI-Table 1: Structure activity relationship of the di-aminopyrimidines. Starvation = starvation induced autophagy assay; Rapamycin = Rapamycin induced autophagy assay; Viability = survival assessed by means of an ADP-glow assay. > 10 = no inhibition at a test concentration of 10 µM. Data is mean; SD = ± standard deviation; n ≥ 3 for all IC_{50} values.

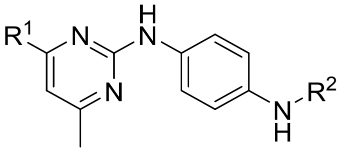

entry	nr.	R¹	R²	Starvation (IC_{50} [µM])	SD [µM] (Starvation)	Rapamycin (IC_{50} [µM])	SD [µM] (Rapamycin)
1	Aumitin (1)	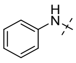	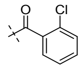	0.12	0.07	0.24	0.20
2	**14**	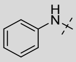	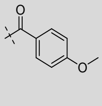	0.37	0.18	1.8	0.9
3	**15**	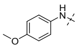	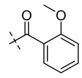	0.38	0.23	1.8	2.0
4	**16**	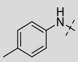	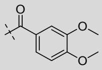	0.73	0.06	3.4	0.8
5	**17**	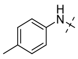	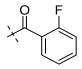	0.75	0.11	1.7	0.5
6	**2**	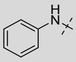	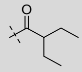	0.80	0.30	0.81	0.68
7	**3**	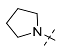	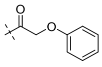	0.81	0.16	0.29	0.18
8	**4**	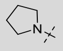	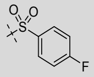	0.97	0.47	0.16	0.08
9	**18**	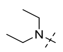	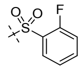	1.1	0.1	1.1	0.2
10	**19**	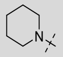	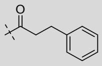	1.3	0.1	na	na
11	**20**	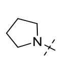	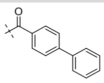	1.3	1.5	na	na
12	**21**	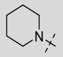	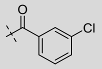	1.5	0.2	4.0	0.3
13	**22**	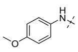	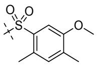	1.6	0.2	3.3	0.8
14	**5**	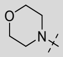	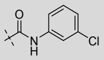	1.6	0.6	4.1	3.0
15	**23**	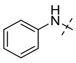	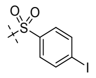	1.8	0.3	3.4	1.7
16	**24**	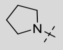	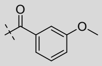	1.8	0.9	na	na

17	**25**	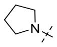	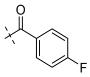	1.9	0.7	0.38	0.05
18	**26**	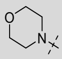	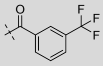	2.0	0.1	2.2	0.2
19	**27**	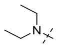	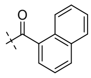	2.0	0.3	3.6	0.5
20	**28**	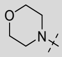	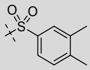	2.0	0.3	1.9	1.1
21	**29**	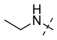	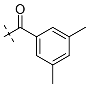	2.1	0.3	5.7	1.7
22	**30**	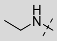	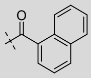	2.2	0.4	1.7	1.1
23	**31**	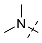	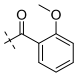	2.3	1.2	1.3	0.7
24	**32**	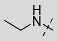	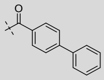	2.4	1.3	2.0	1.7
25	**33**	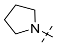	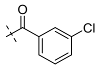	2.4	0.7	1.1	0.3
26	**34**	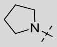	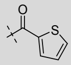	2.5	0.3	2.4	1.0
27	**35**	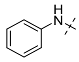	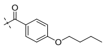	2.7	0.1	1.1	0.5
28	**36**	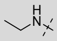	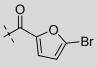	2.8	0.2	3.6	1.1
29	**37**	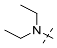	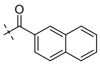	2.8	1.6	3.1	2.1
30	**38**	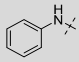	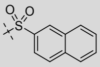	3.0	1.0	3.9	1.3
31	**39**	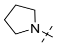	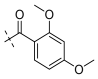	3.1	0.7	1.4	0.6
32	**40**	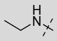	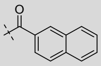	3.5	0.4	3.4	0.7
33	**41**	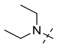	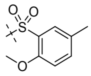	3.6	0.3	7.0	0.8
34	**42**	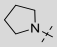	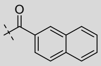	4.0	0.2	2.8	0.3
35	**43**	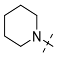	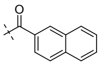	4.1	1.4	5.4	0
36	**44**	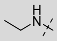	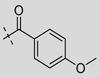	6.9	0.7	2.6	0.9

37	**45**	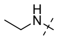	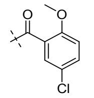	7.0	2.7	na	na
38	**46**	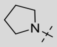	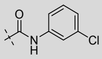	7.8	3.0	2.7	1.1
39	**47**	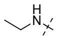	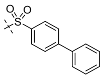	8.6	0.9	na	na
40	**48**	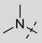	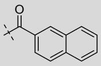	8.6	1.5	na	na
41	**49**	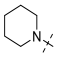	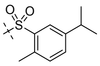	8.8	0.4	3.8	4.1
42	**50**	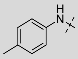	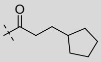	9.5	0.8	4.7	0.3
43	**51**	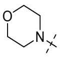	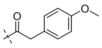	9.4	0	na	na
44	**52**	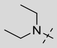	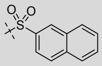	9.9	0	na	na
45	**53**	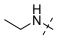	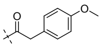	>10	na	na	na
46	**54**	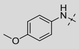	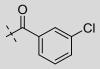	>10	na	na	na
47	**55**	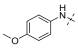	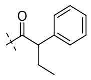	>10	na	na	na
48	**56**	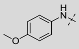	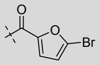	>10	na	na	na
49	**57**	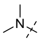	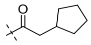	>10	na	na	na
50	**6**	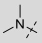	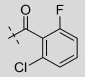	>10	na	na	na
51	**58**	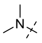	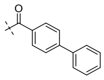	>10	na	na	na
52	**59**	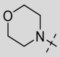	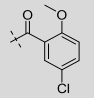	>10	na	na	na
53	**60**	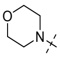	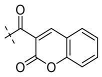	>10	na	na	na
54	**61**	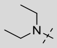	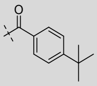	>10	na	na	na
55	**62**	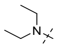	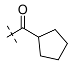	>10	na	na	na
56	**63**	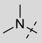	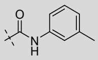	>10	na	na	na

57	**64**	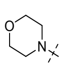	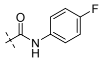	>10	na	na	na
58	**65**	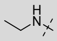	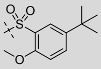	>10	na	9.1	0.7
59	**66**	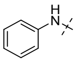	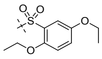	>10	na	na	na
60	**67**	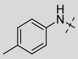	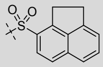	>10	na	na	na
61	**68**	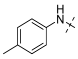	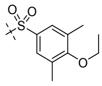	>10	na	na	na
62	**69**	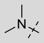	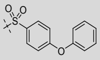	>10	na	na	na
63	**70**	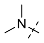	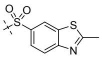	>10	na	na	na
64	**71**	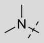	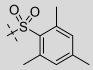	>10	na	na	na
65	**72**	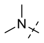	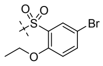	>10	na	na	na
66	**73**	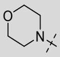	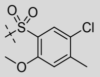	>10	na	na	na
67	**74**	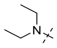	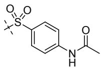	>10	na	na	na
68	**75**	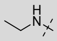	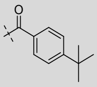	>10	na	na	na
69	**76**	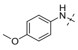	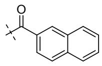	>10	na	na	na
70	**77**	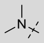	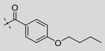	>10	na	3.7	2.2
71	**78**	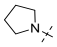	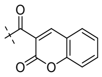	>10	na	na	na
72	**79**	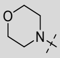	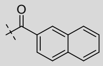	>10	na	0.53	0

SI-Table 2: Biochemical Inhibition of kinases by Aumitin. The detection of biochemical inhibition of overall 419 kinases was carried out by Life Technologies. The screen were performed in three different assays formats: Adapta (activity based), Z-Lyte (activity based) and Lantha (binding based) at a concentration of 1 µM for Aumitin. Only PI4KB was inhibited more than 40 % in the screen and thus tested for in dose response. PIK3C2G was not tested in the initial screen but tested later on directly in dose response.

Method	[ATP] Tested / µM	Kinase Tested	% Inhibition Point 1	% Inhibition Point 2	% Inhibition mean	IC_{50} / nM 1.	IC_{50} / nM 2.
Adapta	10	CAMK1 (CaMK1)	-13	-3	-8		
Adapta	Km app	CDK7/cyclin H/MNAT1	-6	13	3		
Adapta	Km app	CDK9/cyclin T1	5	11	8		
Adapta	Km app	CHUK (IKK alpha)	-8	-12	-10		
Adapta	Km app	DAPK1	32	5	18		
Adapta	Km app	GSG2 (Haspin)	-5	-2	-3		
Adapta	Km app	IRAK1	-17	-12	-15		
Adapta	Km app	LRRK2 FL	-3	12	4		
Adapta	Km app	LRRK2 G2019S FL	-8	7	-1		
Adapta	Km app	LRRK2 G2019S	-5	16	6		
Adapta	Km app	LRRK2 I2020T	-4	-6	-5		
Adapta	Km app	LRRK2 R1441C	-14	3	-5		
Adapta	Km app	LRRK2	-9	10	0		
Adapta	Km app	NUAK1 (ARK5)	0	26	13		
Adapta	10	PI4KA (PI4K alpha)	-1	6	2		
Adapta	Km app	PI4KB (PI4K beta)	43	45	44	301	426
Adapta	Km app	PIK3C2A (PI3K-C2 alpha)	3	3	3		
Adapta	10	PIK3C2B (PI3K-C2 beta)	24	20	22		
Adapta	10	PIK3C2G (PI3K-C2 gamma)	NA	NA	NA	2610	3670
Adapta	Km app	PIK3C3 (hVPS34)	4	23	14		
Adapta	Km app	PIK3CA/PIK3R1 (p110 alpha/p85 alpha)	0	12	6		
Adapta	Km app	PIK3CB/PIK3R1 (p110 beta/p85 alpha)	17	3	10		
Adapta	Km app	PIK3CD/PIK3R1 (p110 delta/p85 alpha)	-6	15	5		
Adapta	Km app	PIK3CG (p110 gamma)	7	4	5		
Adapta	10	PIP4K2A	-1	-3	-2		
Adapta	10	PIP5K1A	11	8	9		
Adapta	10	PIP5K1B	-18	-11	-14		
Adapta	10	PIP5K1C	-3	7	2		
Adapta	Km app	SPHK1	2	-2	0		
Adapta	10	SPHK2	5	-4	1		
Z-Lyte	Km app	ABL1 E255K	8	0	4		
Z-Lyte	Km app	ABL1 G250E	0	-3	-1		
Z-Lyte	Km app	ABL1 T315I	0	1	1		
Z-Lyte	Km app	ABL1 Y253F	5	3	4		
Z-Lyte	Km app	ABL1	19	-1	9		
Z-Lyte	Km app	ABL2 (Arg)	8	6	7		

Z-Lyte	Km app	ACVR1B (ALK4)	11	12	11
Z-Lyte	Km app	ADRBK1 (GRK2)	0	3	2
Z-Lyte	Km app	ADRBK2 (GRK3)	-3	-2	-2
Z-Lyte	Km app	AKT1 (PKB alpha)	-6	-10	-8
Z-Lyte	Km app	AKT2 (PKB beta)	8	12	10
Z-Lyte	Km app	AKT3 (PKB gamma)	12	1	7
Z-Lyte	Km app	ALK	5	-5	0
Z-Lyte	Km app	AMPK A1/B1/G1	4	1	3
Z-Lyte	Km app	AMPK A2/B1/G1	21	12	17
Z-Lyte	Km app	AURKA (Aurora A)	17	16	16
Z-Lyte	Km app	AURKB (Aurora B)	9	7	8
Z-Lyte	Km app	AURKC (Aurora C)	-6	-3	-5
Z-Lyte	Km app	AXL	-6	-4	-5
Z-Lyte	Km app	BLK	18	6	12
Z-Lyte	Km app	BMX	6	3	5
Z-Lyte	100	BRAF V599E	17	13	15
Z-Lyte	100	BRAF	11	5	8
Z-Lyte	Km app	BRSK1 (SAD1)	1	-6	-2
Z-Lyte	Km app	BTK	12	2	7
Z-Lyte	Km app	CAMK1D (CaMKI delta)	6	4	5
Z-Lyte	Km app	CAMK2A (CaMKII alpha)	7	9	8
Z-Lyte	Km app	CAMK2B (CaMKII beta)	1	-2	-1
Z-Lyte	Km app	CAMK2D (CaMKII delta)	6	10	8
Z-Lyte	Km app	CAMK4 (CaMKIV)	-14	-14	-14
Z-Lyte	Km app	CDC42 BPA (MRCKA)	4	-9	-3
Z-Lyte	Km app	CDC42 BPB (MRCKB)	18	3	11
Z-Lyte	Km app	CDK1/cyclin B	4	8	6
Z-Lyte	Km app	CDK2/cyclin A	3	4	4
Z-Lyte	Km app	CDK5/p25	-1	-8	-4
Z-Lyte	Km app	CDK5/p35	0	0	0
Z-Lyte	Km app	CHEK1 (CHK1)	-16	-14	-15
Z-Lyte	Km app	CHEK2 (CHK2)	-15	-5	-10
Z-Lyte	Km app	CLK1	8	8	8
Z-Lyte	Km app	CLK2	6	6	6
Z-Lyte	Km app	CLK3	10	0	5
Z-Lyte	Km app	CSF1R (FMS)	3	2	2
Z-Lyte	Km app	CSK	0	2	1
Z-Lyte	Km app	CSNK1A1 (CK1 alpha 1)	9	8	9
Z-Lyte	Km app	CSNK1D (CK1 delta)	2	4	3
Z-Lyte	Km app	CSNK1E (CK1 epsilon)	6	6	6
Z-Lyte	Km app	CSNK1G1 (CK1 gamma 1)	-1	1	0
Z-Lyte	Km app	CSNK1G2 (CK1 gamma 2)	1	1	1
Z-Lyte	Km app	CSNK1G3 (CK1 gamma 3)	8	0	4
Z-Lyte	Km app	CSNK2A1 (CK2 alpha 1)	3	6	4

Z-Lyte	Km app	CSNK2A2 (CK2 alpha 2)	3	0	2
Z-Lyte	Km app	DAPK3 (ZIPK)	-10	-1	-6
Z-Lyte	Km app	DCAMKL2 (DCK2)	22	6	14
Z-Lyte	Km app	DNA-PK	6	1	3
Z-Lyte	Km app	DYRK1A	1	0	1
Z-Lyte	Km app	DYRK1B	-2	-2	-2
Z-Lyte	Km app	DYRK3	18	-2	8
Z-Lyte	Km app	DYRK4	4	1	3
Z-Lyte	Km app	EEF2K	1	0	0
Z-Lyte	Km app	EGFR (ErbB1) L858R	-10	-17	-14
Z-Lyte	Km app	EGFR (ErbB1) L861Q	-3	2	-1
Z-Lyte	Km app	EGFR (ErbB1) T790M L858R	-3	-3	-3
Z-Lyte	Km app	EGFR (ErbB1) T790M	7	1	4
Z-Lyte	Km app	EGFR (ErbB1)	3	-2	0
Z-Lyte	Km app	EPHA1	5	7	6
Z-Lyte	Km app	EPHA2	16	3	9
Z-Lyte	Km app	EPHA4	21	5	13
Z-Lyte	Km app	EPHA5	6	2	4
Z-Lyte	Km app	EPHA8	11	6	8
Z-Lyte	Km app	EPHB1	0	4	2
Z-Lyte	Km app	EPHB2	2	2	2
Z-Lyte	Km app	EPHB3	19	9	14
Z-Lyte	Km app	EPHB4	6	4	5
Z-Lyte	Km app	ERBB2 (HER2)	-29	-23	-26
Z-Lyte	Km app	ERBB4 (HER4)	2	-7	-2
Z-Lyte	Km app	FER	-2	-1	-1
Z-Lyte	Km app	FES (FPS)	6	-5	0
Z-Lyte	Km app	FGFR1	9	7	8
Z-Lyte	Km app	FGFR2	-1	-6	-4
Z-Lyte	Km app	FGFR3 K650E	-11	-2	-6
Z-Lyte	Km app	FGFR3	-1	4	2
Z-Lyte	Km app	FGFR4	-8	-9	-8
Z-Lyte	Km app	FGR	14	14	14
Z-Lyte	Km app	FLT1 (VEGFR1)	-3	-7	-5
Z-Lyte	Km app	FLT3 D835Y	26	20	23
Z-Lyte	Km app	FLT3	5	4	4
Z-Lyte	Km app	FLT4 (VEGFR3)	12	6	9
Z-Lyte	Km app	FRAP1 (mTOR)	-14	0	-7
Z-Lyte	Km app	FRK (PTK5)	-1	6	2
Z-Lyte	Km app	FYN	9	8	8
Z-Lyte	Km app	GRK4	7	8	7
Z-Lyte	Km app	GRK5	1	5	3
Z-Lyte	Km app	GRK6	1	2	1
Z-Lyte	Km app	GRK7	2	-3	0

Z-Lyte	Km app	GSK3A (GSK3 alpha)	6	4	5
Z-Lyte	Km app	GSK3B (GSK3 beta)	9	16	12
Z-Lyte	Km app	HCK	7	10	8
Z-Lyte	Km app	HIPK1 (Myak)	11	7	9
Z-Lyte	Km app	HIPK2	1	2	2
Z-Lyte	Km app	HIPK3 (YAK1)	4	-1	1
Z-Lyte	Km app	HIPK4	4	-1	2
Z-Lyte	Km app	IGF1R	-6	-3	-4
Z-Lyte	Km app	IKBKB (IKK beta)	6	1	3
Z-Lyte	Km app	IKBKE (IKK epsilon)	15	4	10
Z-Lyte	Km app	INSR	5	8	6
Z-Lyte	Km app	INSRR (IRR)	8	15	12
Z-Lyte	Km app	IRAK4	-9	-1	-5
Z-Lyte	Km app	ITK	-8	-9	-9
Z-Lyte	Km app	JAK1	-1	-2	-2
Z-Lyte	Km app	JAK2 JH1 JH2 V617F	2	-6	-2
Z-Lyte	Km app	JAK2 JH1 JH2	6	-3	2
Z-Lyte	Km app	JAK2	0	-1	0
Z-Lyte	Km app	JAK3	9	1	5
Z-Lyte	Km app	KDR (VEGFR2)	1	0	0
Z-Lyte	Km app	KIT T670I	-2	-4	-3
Z-Lyte	Km app	KIT	-5	-5	-5
Z-Lyte	Km app	LCK	6	2	4
Z-Lyte	Km app	LTK (TYK1)	-8	0	-4
Z-Lyte	Km app	LYN A	2	2	2
Z-Lyte	Km app	LYN B	19	5	12
Z-Lyte	100	MAP2K1 (MEK1)	-17	7	-5
Z-Lyte	100	MAP2K2 (MEK2)	-16	0	-8
Z-Lyte	100	MAP2K6 (MKK6)	9	5	7
Z-Lyte	100	MAP3K8 (COT)	12	2	7
Z-Lyte	Km app	MAP3K9 (MLK1)	-9	-14	-12
Z-Lyte	Km app	MAP4K2 (GCK)	-10	-6	-8
Z-Lyte	Km app	MAP4K4 (HGK)	11	-5	3
Z-Lyte	Km app	MAP4K5 (KHS1)	15	9	12
Z-Lyte	Km app	MAPK1 (ERK2)	27	10	18
Z-Lyte	100	MAPK10 (JNK3)	6	2	4
Z-Lyte	Km app	MAPK11 (p38 beta)	12	10	11
Z-Lyte	Km app	MAPK12 (p38 gamma)	16	10	13
Z-Lyte	Km app	MAPK13 (p38 delta)	8	12	10
Z-Lyte	Km app	MAPK14 (p38 alpha) Direct	7	6	6
Z-Lyte	100	MAPK14 (p38 alpha)	-2	-11	-6
Z-Lyte	Km app	MAPK3 (ERK1)	4	4	4
Z-Lyte	100	MAPK8 (JNK1)	3	-15	-6
Z-Lyte	100	MAPK9 (JNK2)	-7	-3	-5

Z-Lyte	Km app	MAPKAPK2	23	20	21
Z-Lyte	Km app	MAPKAPK3	1	2	1
Z-Lyte	Km app	MAPKAPK5 (PRAK)	3	1	2
Z-Lyte	Km app	MARK1 (MARK)	1	-2	-1
Z-Lyte	Km app	MARK2	14	12	13
Z-Lyte	Km app	MARK3	-8	9	0
Z-Lyte	Km app	MARK4	-1	-3	-2
Z-Lyte	Km app	MATK (HYL)	-2	-2	-2
Z-Lyte	Km app	MELK	-9	-2	-6
Z-Lyte	Km app	MERTK (cMER)	4	1	3
Z-Lyte	Km app	MET (cMet)	7	13	10
Z-Lyte	Km app	MET M1250T	-4	0	-2
Z-Lyte	Km app	MINK1	-17	-5	-11
Z-Lyte	Km app	MKNK1 (MNK1)	-12	1	-5
Z-Lyte	Km app	MST1R (RON)	-1	-3	-2
Z-Lyte	Km app	MST4	-3	-5	-4
Z-Lyte	Km app	MUSK	-1	4	1
Z-Lyte	Km app	MYLK2 (skMLCK)	-5	-9	-7
Z-Lyte	Km app	NEK1	-16	-12	-14
Z-Lyte	Km app	NEK2	-6	-11	-9
Z-Lyte	Km app	NEK4	-3	13	5
Z-Lyte	Km app	NEK6	-3	-4	-3
Z-Lyte	Km app	NEK7	-8	-3	-6
Z-Lyte	Km app	NEK9	-4	-9	-6
Z-Lyte	Km app	NTRK1 (TRKA)	-3	0	-1
Z-Lyte	Km app	NTRK2 (TRKB)	0	-2	-1
Z-Lyte	Km app	NTRK3 (TRKC)	-3	0	-1
Z-Lyte	Km app	PAK1	-11	3	-4
Z-Lyte	Km app	PAK2 (PAK65)	7	3	5
Z-Lyte	Km app	PAK3	19	-5	7
Z-Lyte	Km app	PAK4	16	3	10
Z-Lyte	Km app	PAK6	-25	-11	-18
Z-Lyte	Km app	PAK7 (KIAA1264)	-13	-3	-8
Z-Lyte	Km app	PASK	0	-2	-1
Z-Lyte	Km app	PDGFRA (PDGFR alpha)	-4	-3	-3
Z-Lyte	Km app	PDGFRA D842V	2	-3	0
Z-Lyte	Km app	PDGFRA T674I	9	6	7
Z-Lyte	Km app	PDGFRA V561D	9	-1	4
Z-Lyte	Km app	PDGFRB (PDGFR beta)	1	2	2
Z-Lyte	Km app	PDK1 Direct	-11	-21	-16
Z-Lyte	100	PDK1	13	13	13
Z-Lyte	Km app	PHKG1	1	-14	-7
Z-Lyte	Km app	PHKG2	0	0	0
Z-Lyte	Km app	PIM1	-8	-21	-15

Z-Lyte	Km app	PIM2	-14	-16	-15
Z-Lyte	Km app	PKN1 (PRK1)	2	-3	-1
Z-Lyte	Km app	PLK1	13	8	11
Z-Lyte	Km app	PLK2	2	7	4
Z-Lyte	Km app	PLK3	-3	0	-2
Z-Lyte	Km app	PRKACA (PKA)	-3	-1	-2
Z-Lyte	Km app	PRKCA (PKC alpha)	0	6	3
Z-Lyte	Km app	PRKCB1 (PKC beta I)	-10	5	-2
Z-Lyte	Km app	PRKCB2 (PKC beta II)	-12	-6	-9
Z-Lyte	Km app	PRKCD (PKC delta)	-14	-9	-12
Z-Lyte	Km app	PRKCE (PKC epsilon)	-12	-8	-10
Z-Lyte	Km app	PRKCG (PKC gamma)	-2	-18	-10
Z-Lyte	Km app	PRKCH (PKC eta)	-2	1	0
Z-Lyte	Km app	PRKCI (PKC iota)	-2	-1	-1
Z-Lyte	Km app	PRKCN (PKD3)	10	9	10
Z-Lyte	Km app	PRKCQ (PKC theta)	5	-7	-1
Z-Lyte	Km app	PRKCZ (PKC zeta)	8	3	6
Z-Lyte	Km app	PRKD1 (PKC mu)	2	-1	0
Z-Lyte	Km app	PRKD2 (PKD2)	-6	-3	-5
Z-Lyte	Km app	PRKG1	-2	0	-1
Z-Lyte	Km app	PRKG2 (PKG2)	1	-1	0
Z-Lyte	Km app	PRKX	12	0	6
Z-Lyte	Km app	PTK2 (FAK)	6	6	6
Z-Lyte	Km app	PTK2B (FAK2)	10	0	5
Z-Lyte	Km app	PTK6 (Brk)	7	7	7
Z-Lyte	100	RAF1 (cRAF) Y340D Y341D	18	5	12
Z-Lyte	Km app	RET V804L	4	5	4
Z-Lyte	Km app	RET Y791F	6	9	8
Z-Lyte	Km app	RET	7	7	7
Z-Lyte	Km app	ROCK1	20	7	14
Z-Lyte	Km app	ROCK2	9	17	13
Z-Lyte	Km app	ROS1	1	-1	0
Z-Lyte	Km app	RPS6KA1 (RSK1)	3	8	5
Z-Lyte	Km app	RPS6KA2 (RSK3)	0	6	3
Z-Lyte	Km app	RPS6KA3 (RSK2)	7	9	8
Z-Lyte	Km app	RPS6KA4 (MSK2)	0	4	2
Z-Lyte	Km app	RPS6KA5 (MSK1)	5	6	6
Z-Lyte	Km app	RPS6KA6 (RSK4)	-4	1	-2
Z-Lyte	Km app	RPS6KB1 (p70S6K)	-5	1	-2
Z-Lyte	Km app	SGK (SGK1)	2	5	4
Z-Lyte	Km app	SGK2	-7	8	1
Z-Lyte	Km app	SGKL (SGK3)	-2	4	1
Z-Lyte	Km app	SNF1LK2	15	0	7
Z-Lyte	Km app	SRC N1	15	11	13

Z-Lyte	Km app	SRC	21	7	14
Z-Lyte	Km app	SRMS (Srm)	2	4	3
Z-Lyte	Km app	SRPK1	1	4	2
Z-Lyte	Km app	SRPK2	-12	-13	-13
Z-Lyte	Km app	STK22B (TSSK2)	6	5	5
Z-Lyte	Km app	STK22D (TSSK1)	-2	0	-1
Z-Lyte	Km app	STK23 (MSSK1)	0	4	2
Z-Lyte	Km app	STK24 (MST3)	-3	-2	-2
Z-Lyte	Km app	STK25 (YSK1)	8	-10	-1
Z-Lyte	Km app	STK3 (MST2)	-6	-9	-8
Z-Lyte	Km app	STK4 (MST1)	-8	-7	-7
Z-Lyte	Km app	SYK	1	-1	0
Z-Lyte	Km app	TAOK2 (TAO1)	2	-6	-2
Z-Lyte	Km app	TBK1	-4	-10	-7
Z-Lyte	Km app	TEK (Tie2)	10	14	12
Z-Lyte	Km app	TXK	2	-1	1
Z-Lyte	Km app	TYK2	-7	-6	-6
Z-Lyte	Km app	TYRO3 (RSE)	7	3	5
Z-Lyte	Km app	YES1	13	10	11
Z-Lyte	Km app	ZAP70	5	2	3
Lantha	NA	ABL1 H396P	1	1	1
Lantha	NA	ABL1 M351T	4	10	7
Lantha	NA	ABL1 Q252H	-2	-2	-2
Lantha	NA	ACVR1 (ALK2) R206H	-11	-22	-16
Lantha	NA	ACVR1 (ALK2)	-1	0	-1
Lantha	NA	ACVR2A	4	0	2
Lantha	NA	ACVR2B	5	8	7
Lantha	NA	ACVRL1 (ALK1)	0	-6	-3
Lantha	NA	ALK C1156Y	12	8	10
Lantha	NA	ALK F1174L	2	3	3
Lantha	NA	ALK L1196M	-2	5	2
Lantha	NA	ALK R1275Q	1	1	1
Lantha	NA	AMPK (A1/B1/G2)	0	-7	-4
Lantha	NA	AMPK (A1/B1/G3)	2	-3	0
Lantha	NA	AMPK (A1/B2/G1)	1	-1	0
Lantha	NA	AMPK (A2/B2/G1)	1	10	5
Lantha	NA	AMPK (A2/B2/G2)	1	2	1
Lantha	NA	AXL R499C	3	3	3
Lantha	NA	BMPR1A (ALK3)	1	-2	-1
Lantha	NA	BMPR1B (ALK6)	1	-9	-4
Lantha	NA	BMPR2	-6	-3	-5
Lantha	NA	BRAF V599E	-5	1	-2
Lantha	NA	BRAF	-2	7	3
Lantha	NA	BRSK2	5	4	5

Lantha	NA	CAMK2G (CaMKII gamma)	-1	5	2
Lantha	NA	CAMKK1 (CAMKKA)	-10	-5	-8
Lantha	NA	CAMKK2 (CaMKK beta)	-1	2	1
Lantha	NA	CASK	-10	3	-4
Lantha	NA	CDC7/DBF4	0	0	0
Lantha	NA	CDK1/cyclin A2	-8	4	-2
Lantha	NA	CDK11 (Inactive)	14	19	17
Lantha	NA	CDK14 (PFTK1)/cyclin Y	6	2	4
Lantha	NA	CDK16 (PCTK1)/cyclin Y	11	3	7
Lantha	NA	CDK2/cyclin A1	0	4	2
Lantha	NA	CDK2/cyclin E1	-2	-1	-1
Lantha	NA	CDK2/cyclin O	-2	2	0
Lantha	NA	CDK3/cyclin E1	4	9	6
Lantha	NA	CDK5 (Inactive)	-1	8	3
Lantha	NA	CDK8/cyclin C	-1	-3	-2
Lantha	NA	CDK9 (Inactive)	11	-3	4
Lantha	NA	CDK9/cyclin K	1	1	1
Lantha	NA	CLK4	-3	3	0
Lantha	NA	DAPK2	0	1	0
Lantha	NA	DDR1	-1	-1	-1
Lantha	NA	DDR2 N456S	4	-3	0
Lantha	NA	DDR2 T654M	-7	0	-3
Lantha	NA	DDR2	-1	-5	-3
Lantha	NA	DMPK	12	5	9
Lantha	NA	DYRK2	13	3	8
Lantha	NA	EGFR (ErbB1) d746-750	-2	-1	-2
Lantha	NA	EIF2AK2 (PKR)	-6	-4	-5
Lantha	NA	EPHA3	-4	2	-1
Lantha	NA	EPHA6	-10	3	-4
Lantha	NA	EPHA7	-4	-9	-6
Lantha	NA	FGFR1 V561M	-1	-5	-3
Lantha	NA	FGFR3 G697C	-6	-1	-3
Lantha	NA	FGFR3 K650M	-4	5	0
Lantha	NA	FLT3 ITD	-1	-4	-2
Lantha	NA	FYN A	3	5	4
Lantha	NA	GRK1	2	-1	1
Lantha	NA	ICK	0	2	1
Lantha	NA	KIT A829P	1	1	1
Lantha	NA	KIT D816H	-3	2	0
Lantha	NA	KIT D816V	2	2	2
Lantha	NA	KIT D820E	2	2	2
Lantha	NA	KIT N822K	-1	-1	-1
Lantha	NA	KIT T670E	1	-4	-1
Lantha	NA	KIT V559D T670I	3	2	3

Lantha	NA	KIT V654A	-4	2	-1
Lantha	NA	KIT Y823D	0	6	3
Lantha	NA	LATS1	2	-6	-2
Lantha	NA	LATS2	-3	-5	-4
Lantha	NA	LIMK1	1	-2	0
Lantha	NA	LIMK2	-6	-4	-5
Lantha	NA	MAP2K1 (MEK1) S218D S222D	-4	2	-1
Lantha	NA	MAP2K1 (MEK1)	-3	-3	-3
Lantha	NA	MAP2K2 (MEK2)	-3	1	-1
Lantha	NA	MAP2K3 (MEK3)	4	7	5
Lantha	NA	MAP2K6 (MKK6) S207E T211E	-2	-4	-3
Lantha	NA	MAP2K6 (MKK6)	1	1	1
Lantha	NA	MAP3K10 (MLK2)	1	2	1
Lantha	NA	MAP3K11 (MLK3)	-1	1	0
Lantha	NA	MAP3K14 (NIK)	-3	-3	-3
Lantha	NA	MAP3K2 (MEKK2)	4	-4	0
Lantha	NA	MAP3K3 (MEKK3)	-2	6	2
Lantha	NA	MAP3K5 (ASK1)	-10	-1	-6
Lantha	NA	MAP3K7/MAP3K7IP1 (TAK1-TAB1)	-3	2	-1
Lantha	NA	MAP4K1 (HPK1)	0	-1	0
Lantha	NA	MAP4K3 (GLK)	-4	3	0
Lantha	NA	MAPK10 (JNK3)	5	6	6
Lantha	NA	MAPK15 (ERK7)	-2	-2	-2
Lantha	NA	MAPK8 (JNK1)	10	4	7
Lantha	NA	MAPK9 (JNK2)	5	3	4
Lantha	NA	MERTK (cMER) A708S	3	-2	1
Lantha	NA	MET D1228H	3	1	2
Lantha	NA	MKNK2 (MNK2)	1	-1	0
Lantha	NA	MLCK (MLCK2)	-4	-3	-3
Lantha	NA	MYLK (MLCK)	-2	-2	-2
Lantha	NA	MYO3B (MYO3 beta)	0	2	1
Lantha	NA	NLK	2	6	4
Lantha	NA	NUAK2	1	0	1
Lantha	NA	PKN2 (PRK2)	-4	-3	-4
Lantha	NA	PLK4	2	5	3
Lantha	NA	PRKACB (PRKAC beta)	5	1	3
Lantha	NA	PRKACG (PRKAC gamma)	-1	-3	-2
Lantha	NA	RAF1 (cRAF) Y340D Y341D	-2	0	-1
Lantha	NA	RET G691S	1	7	4
Lantha	NA	RET M918T	2	-8	-3
Lantha	NA	RET V804M	15	23	19
Lantha	NA	RIPK2	0	2	1
Lantha	NA	RIPK3	0	0	0
Lantha	NA	SIK1	0	0	0

Lantha	NA	SIK3	6	9	8
Lantha	NA	SLK	-1	-3	-2
Lantha	NA	STK16 (PKL12)	1	3	2
Lantha	NA	STK17A (DRAK1)	12	7	9
Lantha	NA	STK17B (DRAK2)	1	2	2
Lantha	NA	STK32B (YANK2)	0	3	1
Lantha	NA	STK32C (YANK3)	4	3	3
Lantha	NA	STK33	0	-2	-1
Lantha	NA	STK38 (NDR)	-2	7	3
Lantha	NA	STK38L (NDR2)	4	7	6
Lantha	NA	STK39 (STLK3)	-2	8	3
Lantha	NA	TAOK1	14	19	17
Lantha	NA	TAOK3 (JIK)	3	-1	1
Lantha	NA	TEC	-3	-3	-3
Lantha	NA	TEK (TIE2) R849W	4	3	4
Lantha	NA	TEK (TIE2) Y1108F	-1	-3	-2
Lantha	NA	TESK2	9	11	10
Lantha	NA	TGFBR1 (ALK5)	-5	-2	-4
Lantha	NA	TGFBR2	1	-4	-1
Lantha	NA	TLK1	-1	-4	-2
Lantha	NA	TLK2	-2	-3	-3
Lantha	NA	TNIK	-3	-6	-4
Lantha	NA	TNK2 (ACK)	1	1	1
Lantha	NA	TTK	0	-4	-2
Lantha	NA	ULK1	3	-3	0
Lantha	NA	ULK2	4	3	4
Lantha	NA	ULK3	8	0	4
Lantha	NA	WEE1	1	-1	0
Lantha	NA	WNK2	9	3	6
Lantha	NA	WNK3	6	7	6
Lantha	NA	ZAK	0	0	0

SI-Table 3: Examination of the off-target effects of Aumitin by the employment of literature reported kinase inhibitors. Bought or synthesized literature reported kinase inhibitors, their known targets and their effect in our autophagy assays are shown. The biochemical kinase inhibition was measured in two independent experiments, with each datapoint in duplicate. Starvation = starvation induced autophagy assay; Rapamycin = Rapamycin induced autophagy assay. Inactive = no inhibition at a test concentration > 10 µM. Data is mean ± SD, n ≥ 3 for all autophagy inhibition values.

Compound name	Structure	Kinase targets		Autophagy Inhibition	
		Kinase name	Biochemical inhibition IC_{50} [nM]	Starvation IC_{50} [µM]	Rapamycin IC_{50} [µM]
Aumitin (1)	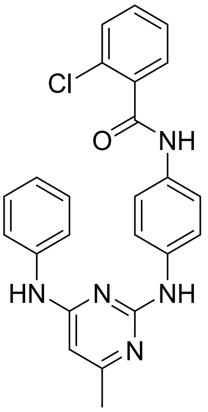	PI4KB<br>PIK3C2G	301, 426<br>2610, 3670	0.124 ± 0.070	0.244 ± 0.200
PI4KB inhibitor 1[1]	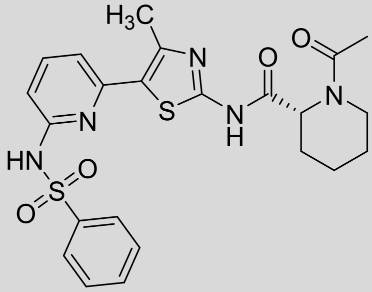	PI4KB	see literature	Inactive	Inactive
PI4KB inhibitor 2[2]	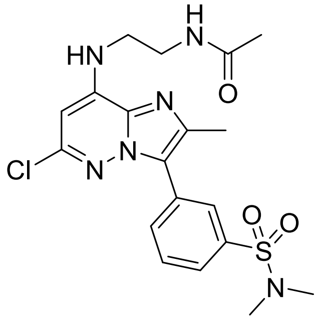	PI4KB	see literature	Inactive	Inactive
PIK3C2g inhibitor 1[3,4]	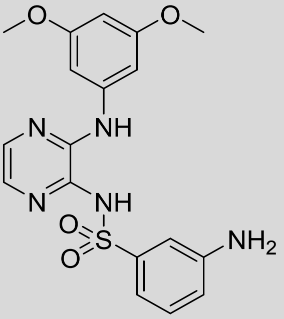	PIK3C2g	see literature	Inactive	Inactive

PIK3C2g inhibitor 2[3,4]	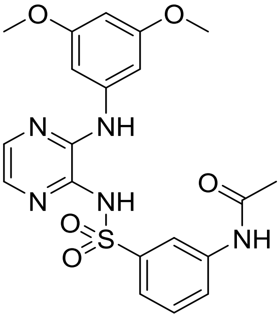	PIK3C2g	see literature	Inactive	Inactive
PIK3C2g inhibitor 3[3,4]	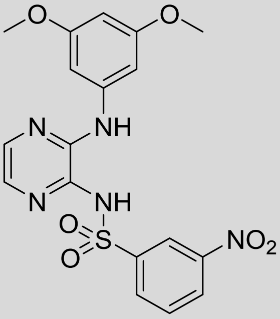	PIK3C2g	see literature	2.40 ± 1.50	Inactive
PIK3C2g inhibitor 4[3,4]	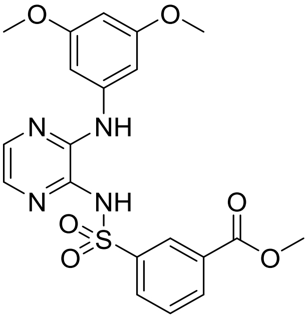	PIK3C2g	see literature	Inactive	Inactive

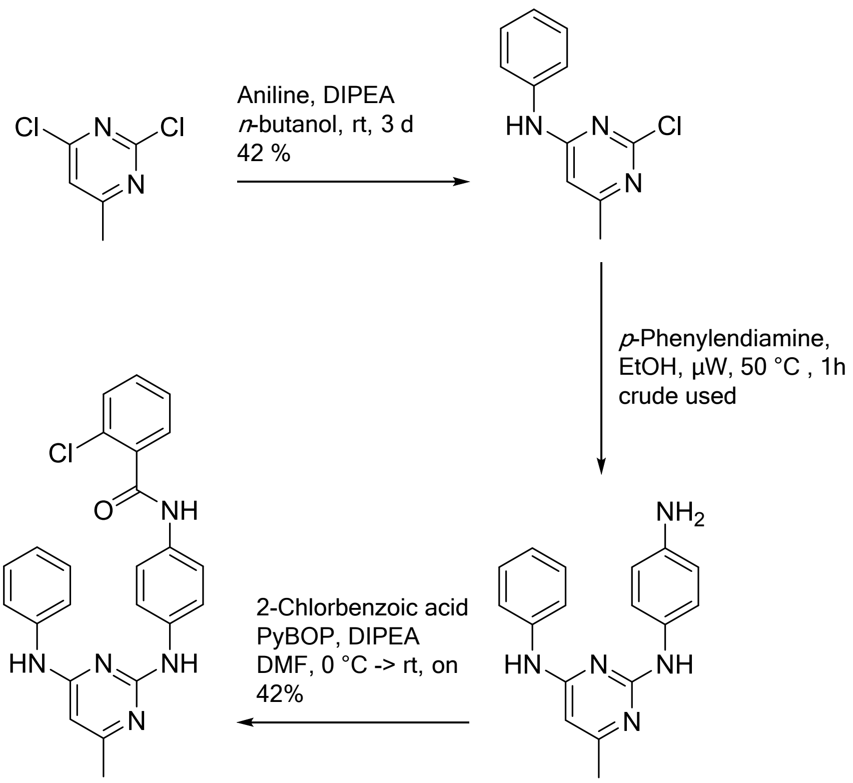

SI-Figure 1: Synthesis of Aumitin. The regioselective replacement of the 4-chloride substituent in the first reaction was achieved by changing the solvent to *n*-butanol. The synthesis of Aumitin started from commercially available 2,4-dichloro-6-methylpyrimidine, which was regioselectively reacted with aniline to replace the 4-chloro substituent. A screen of different bases and solvents revealed *n*-butanol as the superior solvent for the employment of DIPEA as the base. The second nucleophilic substitution on the pyrimidine occurred neatly with *p*-phenylendiamine in ethanol in the microwave. The amide-coupling succeeded following standard conditions using PyBOP.

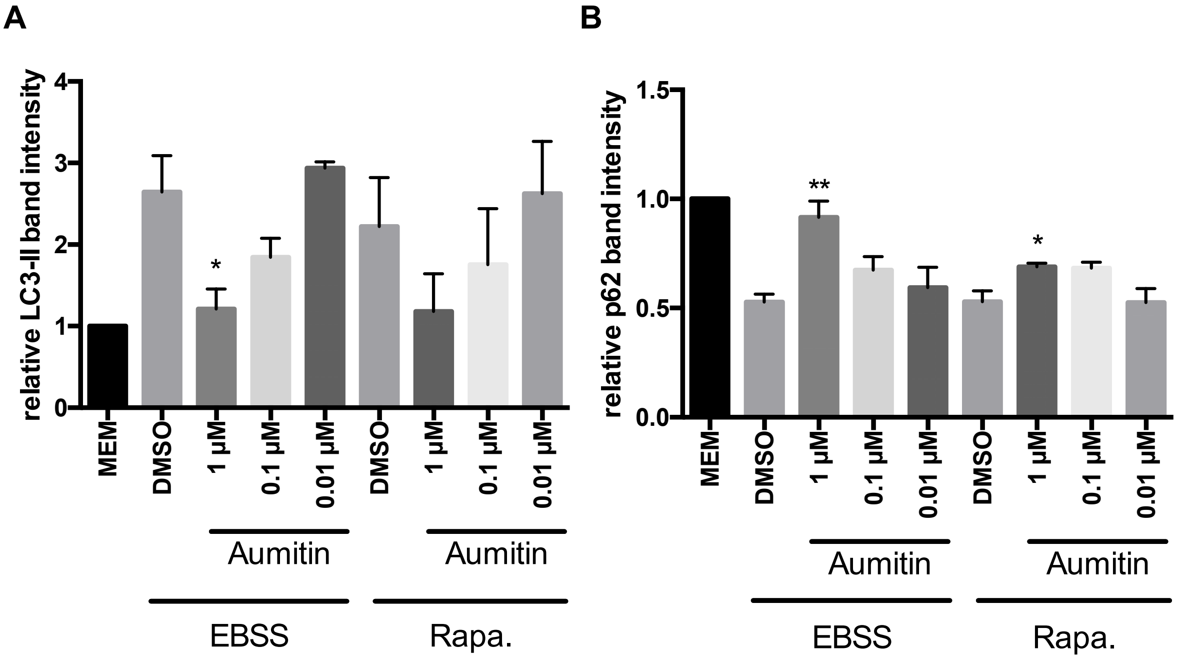

SI-Figure 2: Quantification of LC3 and p62 blots from Figure 1F. **A:** Relative band intensities for LC3-II normalized to the MEM (DMSO) control. Aumitin significantly inhibited LC3 lipidation to LC3-II compared to the EBSS (DMSO) control ($p = 0.048$, students t-test). **B:** Relative band intensities for p62 normalized to the MEM (DMSO) control. Aumitin significantly inhibited p62 degradation compared to the DMSO control ($p = 0.009$ for EBSS induced autophagy, and $p = 0.04$ for rapamycin-induced autophagy, students t-test). All data the mean ± sem (n=3).

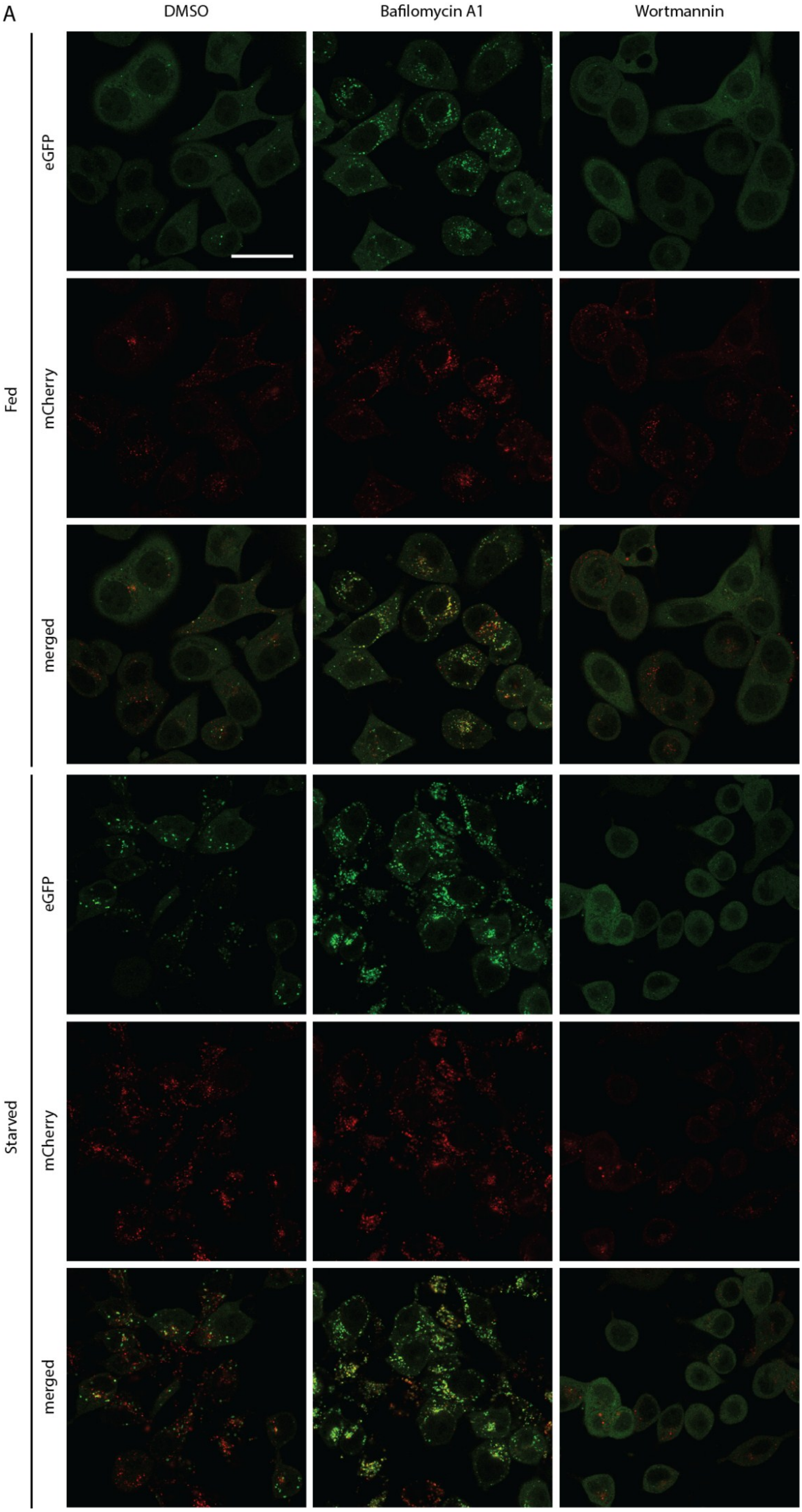
A
DMSO
Bafilomycin A1
Wortmannin
Fed
eGFP
mCherry
merged
Starved
eGFP
mCherry
merged

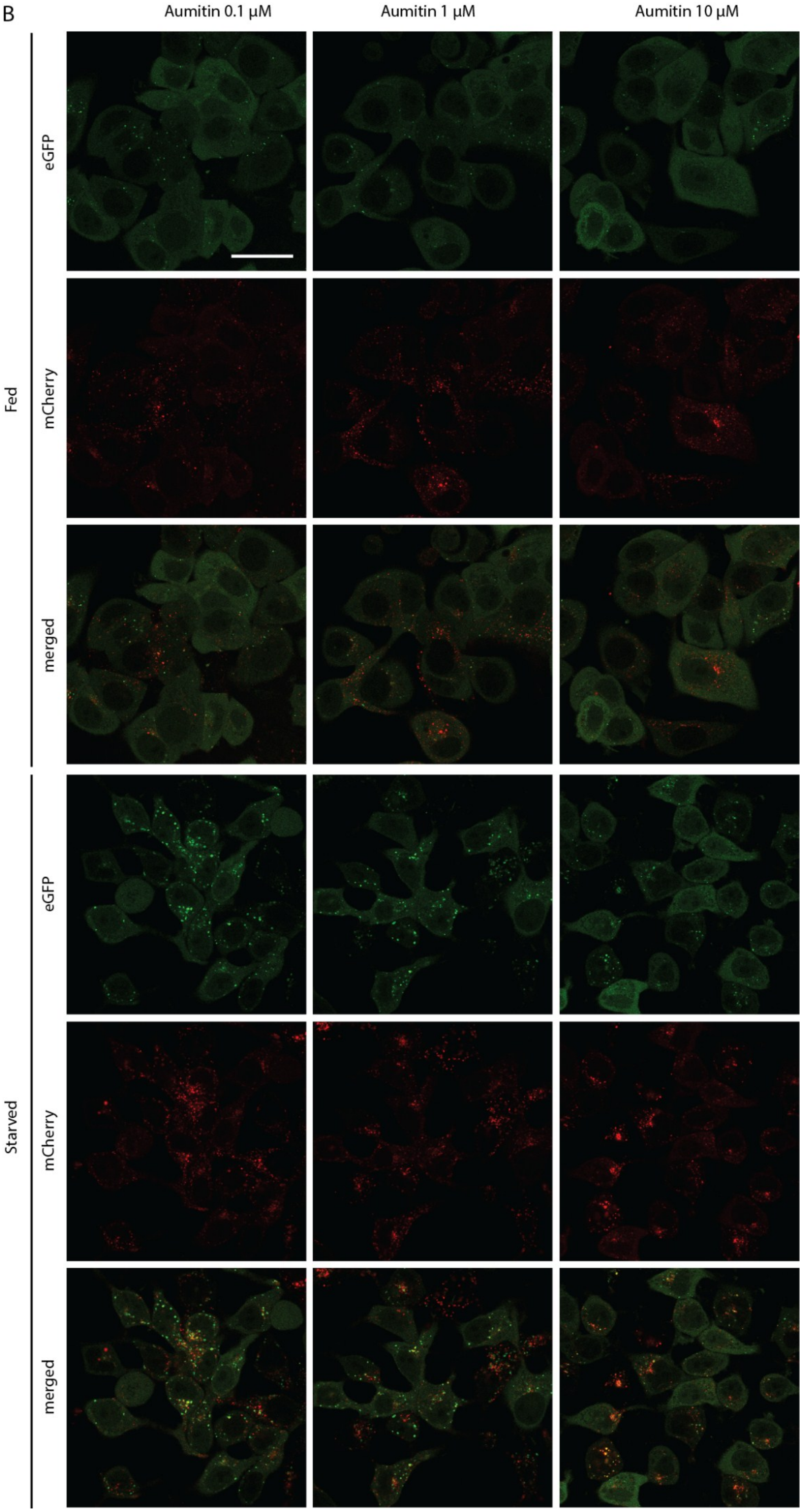
B
Aumitin 0.1 µM
Aumitin 1 µM
Aumitin 10 µM
Fed
eGFP
mCherry
merged
Starved
eGFP
mCherry
merged

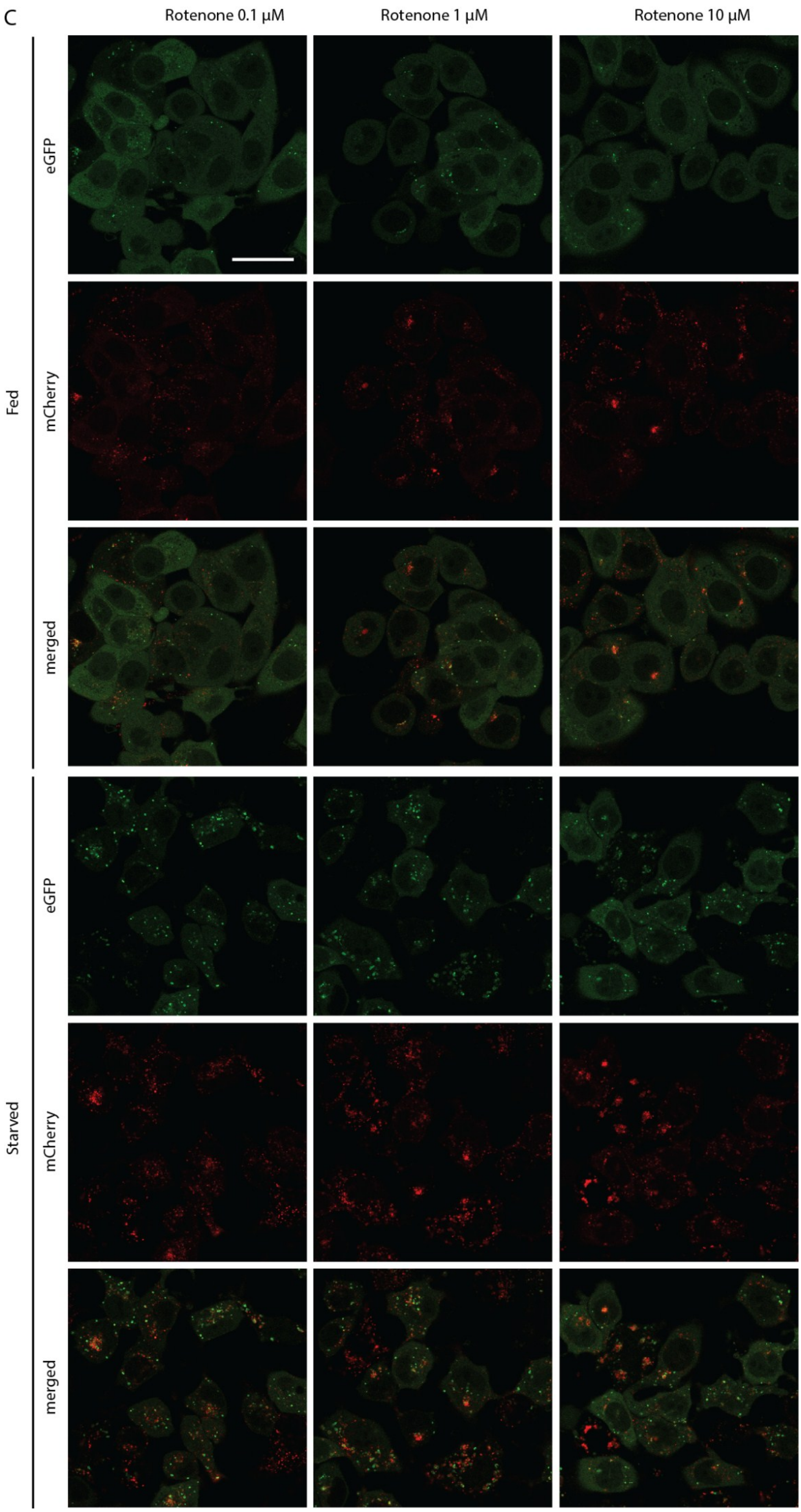
C
Rotenone 0.1 µM
Rotenone 1 µM
Rotenone 10 µM
Fed
eGFP
mCherry
merged
Starved
eGFP
mCherry
merged

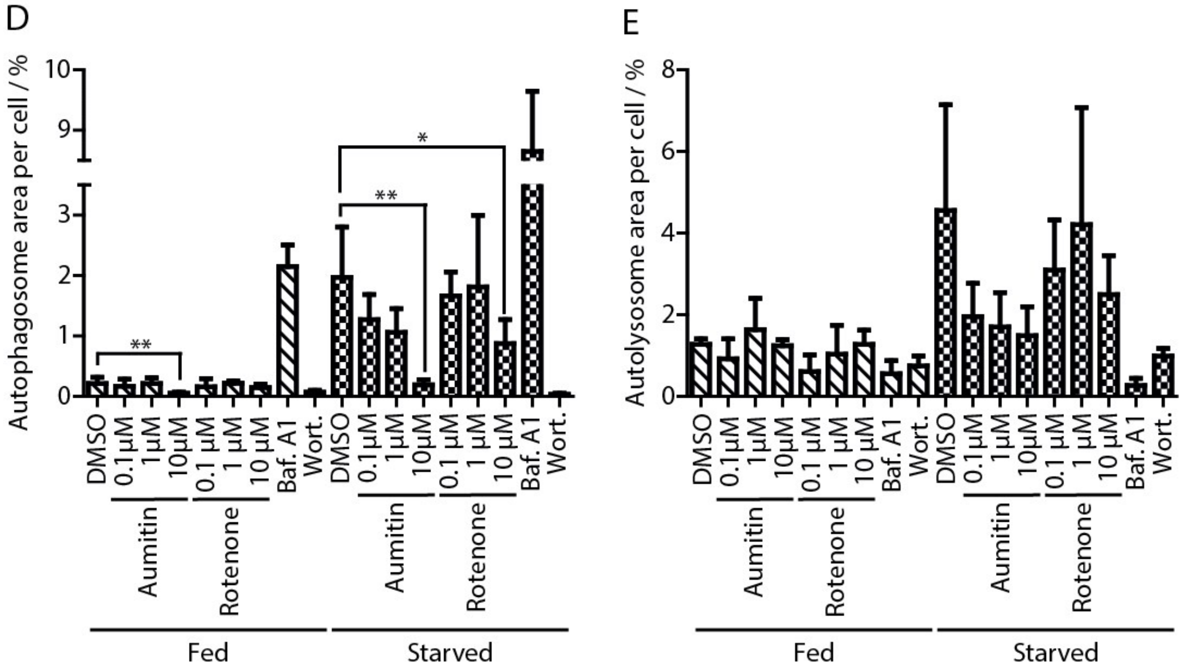

SI-Figure 3: Aumitin and Rotenone inhibit autophagosome accumulation but not autolysosome accumulation. **A:** Fluorescence microscopy images of fed and starved MCF7 cells stably expressing mCherry-eGFP-LC3 for DMSO, Bafilomycin A1 (Baf. A1, 50 nM) and Wortmannin (Wort., 500 nM). **B:** Fluorescence microscopy images for different concentrations of Aumitin. **C:** Fluorescence microscopy images for different concentrations of Rotenone. **D:** Quantification of eGFP puncta showed a dose-dependent reduction of autophagosomes under fed as well as under starved conditions by Aumitin (fed, 10 µM, P = 0.0073; starved, 10 µM, P = 0.0014) and Rotenone (starved, 10 µM, P = 0.0285), similarly to the known autophagy inhibitor Wortmannin and the opposite effect of Bafilomycin A1. **E:** Quantification of mCherry puncta for Aumitin and Rotenone on the accumulation of autolysosomes. Taken together it shows that these complex I inhibitors do not appear to be inhibitors of the fusion of autophagosomes with lysosomes but inhibitors of the formation of autophagosomes. Scale bar = 20 µm. **Statistical analysis was performed using student's t-test (* = P ≤ 0.05; ** = P ≤ 0.005). Data are shown with mean values ± SD.**

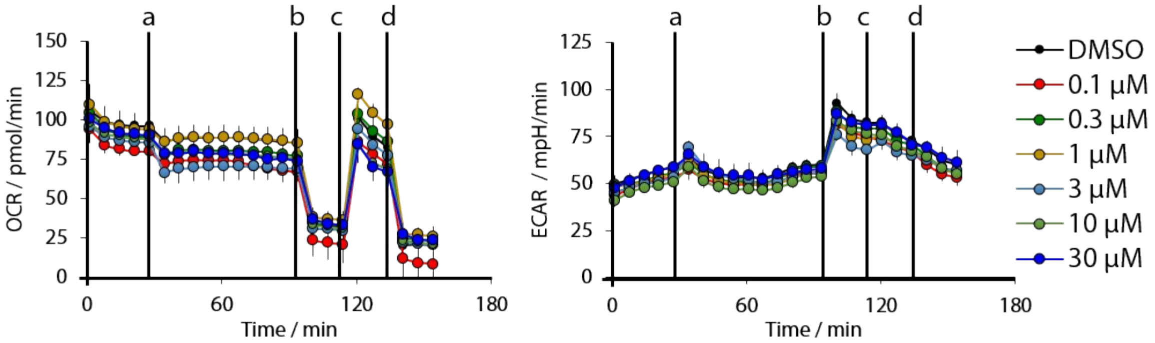

SI-Figure 4: Influence Autophinib on mitochondrial respiration. HeLa cells were treated with the respective compound in a Seahorse XFe96 Analyzer and oxygen consumption rate (OCR) and extracellular acidification rate (ECAR) were measured dose and time dependently. At timepoint a) the test compound, in this case the autophagy inhibitor was added to the cells. At timepoint b) Oligomycin was added at a concentration of 1 µM. At timepoint c) the decoupling agent FCCP was added in a concentration of 125 nM. At timepoint d) Rotenone and Antimycin were both added to the samples both at a concentration of 1 µM each. All data is mean ± SD, n = 2, representative experiments shown.

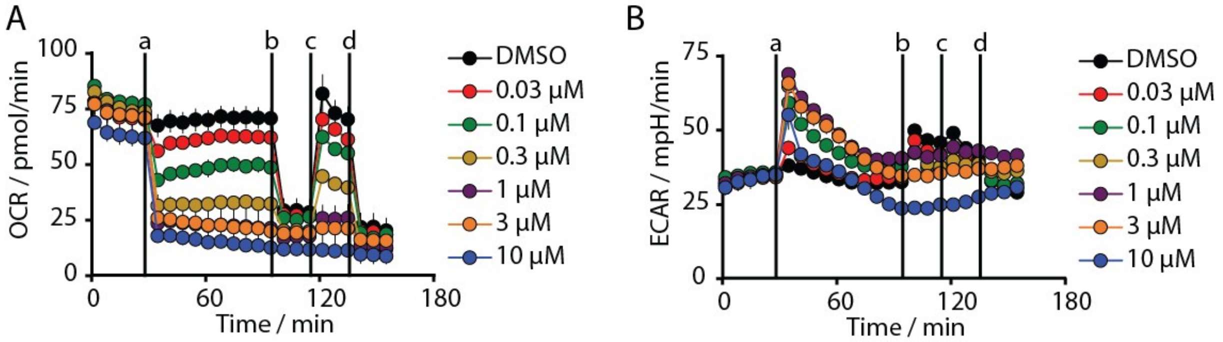

SI-Figure 5: Influence of autophagy inhibitors on mitochondrial respiration in HeLa cells. HeLa cells were treated with the respective compound in a Seahorse device and oxygen consumption rate (OCR) and extracellular acidification rate (ECAR) were measured dose and time dependently. At timepoint a Aumitin was added to the cells. At timepoint b Oligomycin was added at a concentration of 1 µM. At timepoint c the decoupling agent FCCP was added in a concentration of 125 nM. At timepoint d Rotenone and Antimycin were both added to the samples both at a concentration of 1 µM each. **A:** OCR measured for Aumitin in HeLa cells. **B:** ECAR measured for Aumitin in HeLa cells. All data is mean ± SD, n = 3, representative experiments shown.

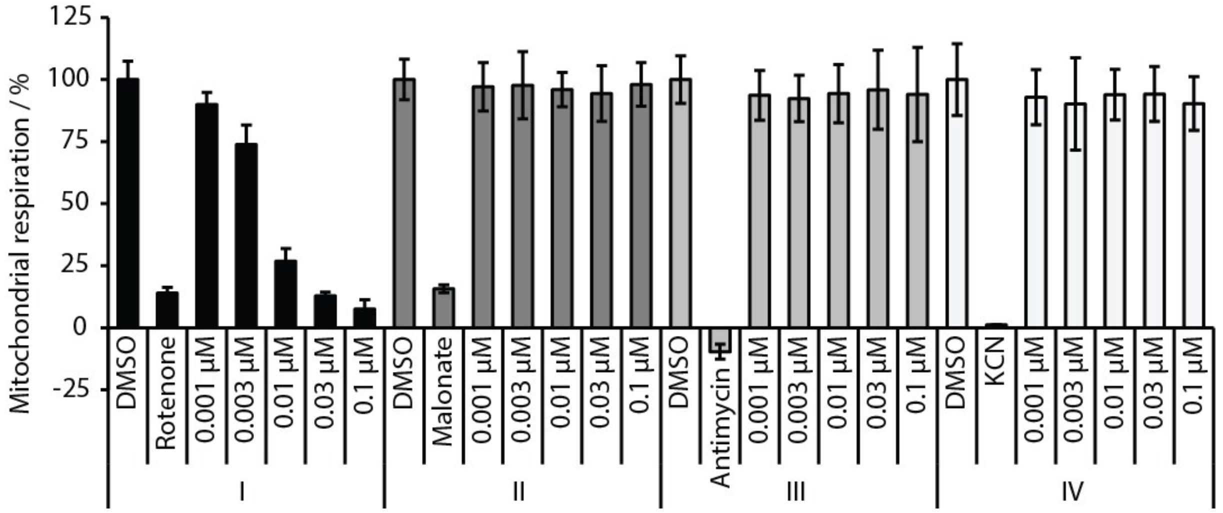

SI-Figure 6: Semi-intact assay performed for Rotenone. HeLa cells were semi-permeabilized by digitonin and treated with the specific substrates of each mitochondrial complex. Rotenone only inhibited the pyruvate/malate-driven respiratory activity of the NADH-CoQ reductase activity but did not inhibit the enzymatic activity of any following complex.

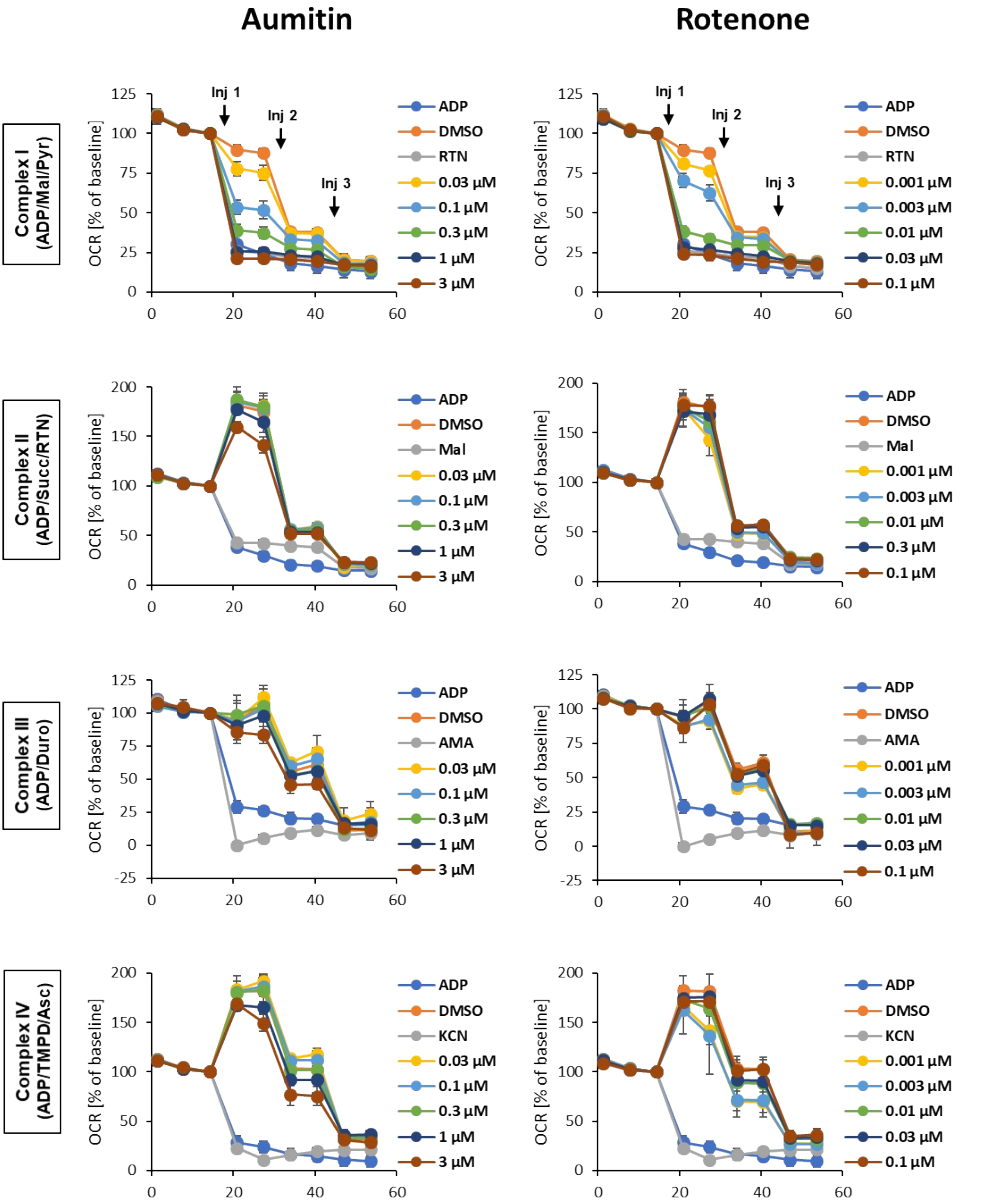

SI-Figure 7: The effect of Aumitin/Rotenone on each complex activity.

The respiration activity of each complex was determined as follows: Samples were co-injected with ADP, substrates, and digitonin (inj 1). Note that the following reagents were used for positive controls for each activity test; rotenone (RTN; 0.03 µM), malonate (Mal; 2 mM), antimycin A (AMA; 0.03 µM), and KCN (20 mM) (n = 3). The state 3 OCR and the mitochondrial respiration were calculated using the following formula; (state 3 OCR) = (measurement #4)- (measurement #8), and the mitochondrial respiration (%) = $(\text{state 3 OCR})_{sample}$ / $(\text{state 3 OCR})_{DMSO}$ x100.

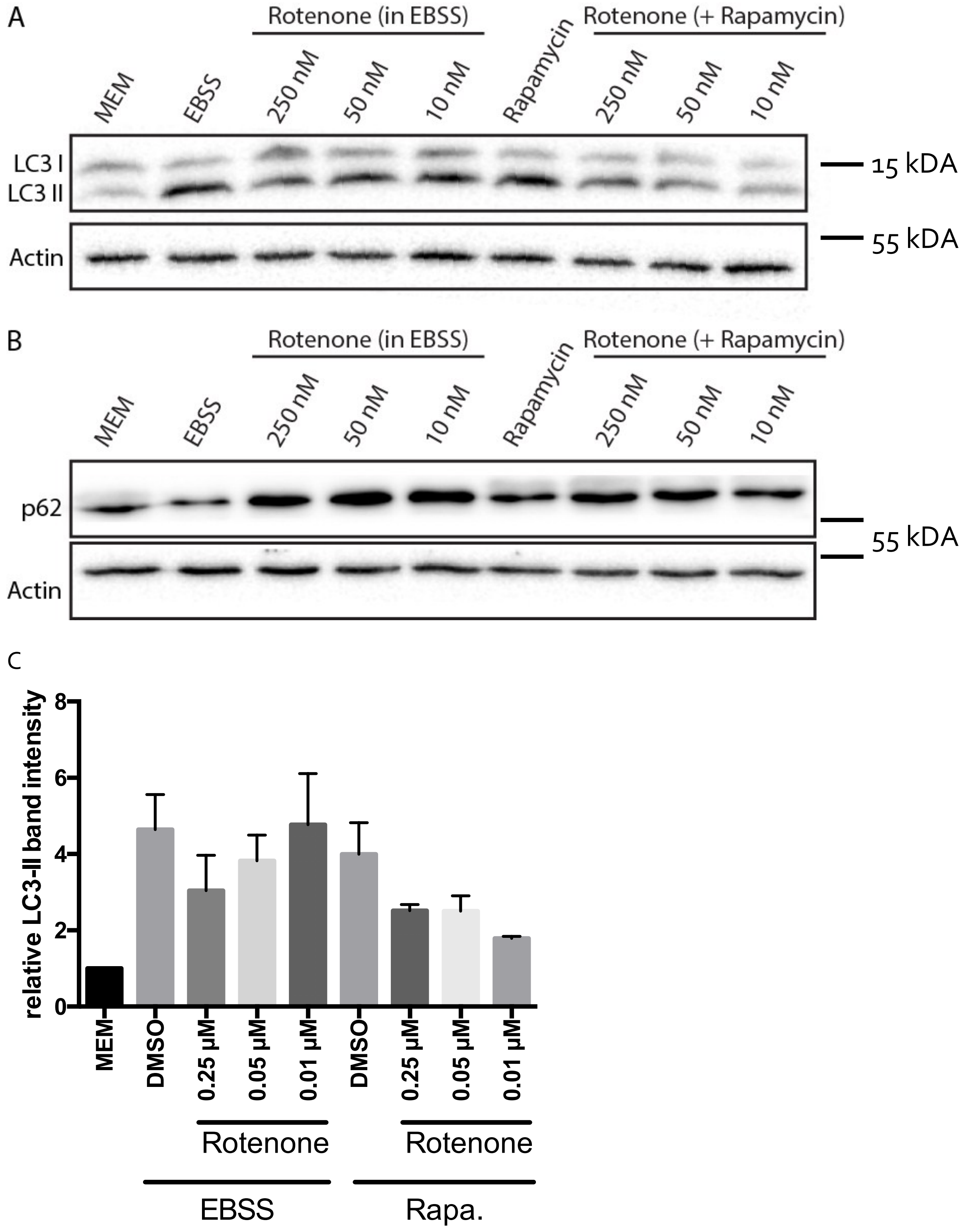

SI-Figure 8: Inhibition of LC3-II lipidation and p62 degradation by Rotenone in MCF7-eGFP-LC3 cells. Starvation as well as Rapamycin (Rapa. 100 nM) induced autophagy induces lipidation of LC3-I to LC3-II and degradation of p62. **A:** Rotenone inhibits LC3 lipidation to LC3-II for starvation- as well as Rapamycin-induced autophagy. **B:** Rotenone inhibits p62 degradation for starvation- as well as Rapamycin-induce autophagy. **C:** Quantification of LC3-II band intensity for n ≥ 3 blots. Band intensity was normalized to the MEM (DMSO) control. Representative blots shown.

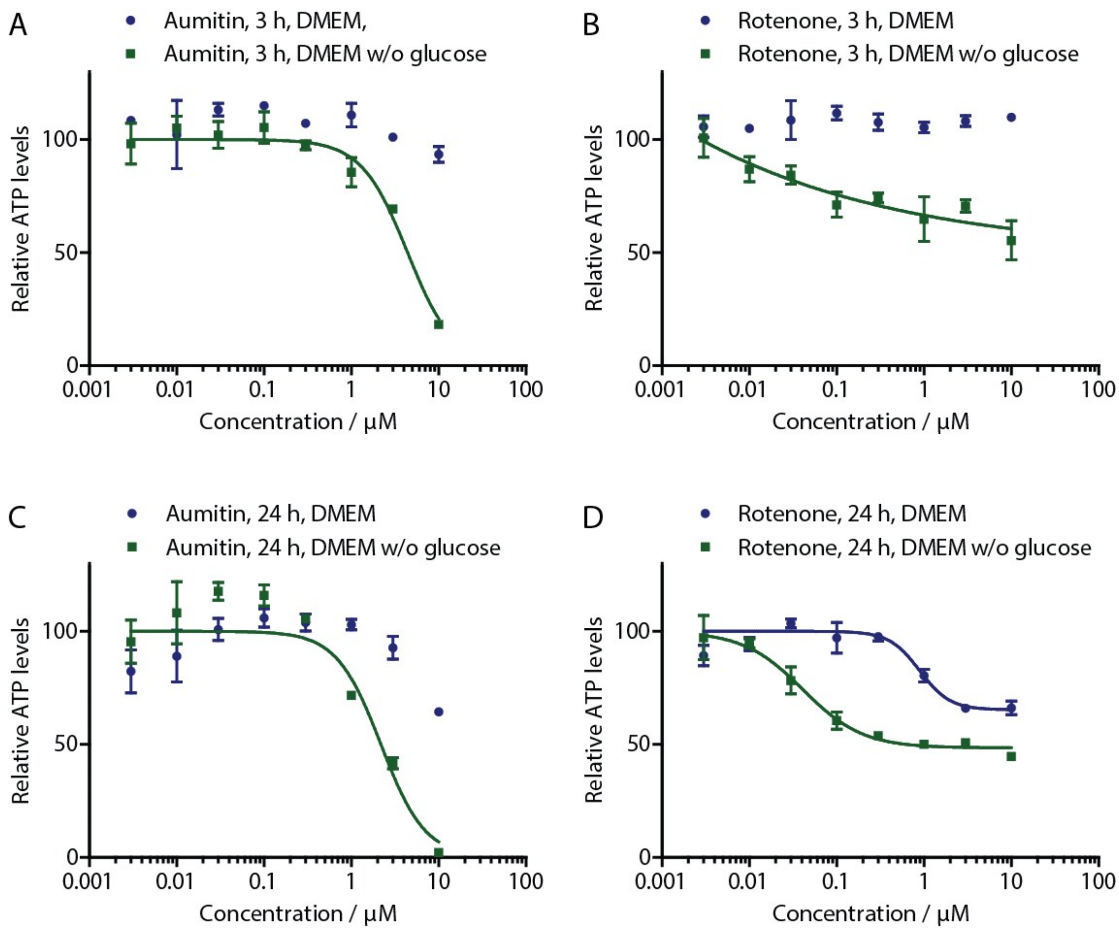

SI-Figure 9: Selective viability of MCF7 cells upon pharmacological mitochondrial complex I inhibition. Relative ATP levels were determined by means of a CellTiter-Glo® assay and normalized to the ATP levels measured for DMSO treated cells. The ATP levels are considered to be proportional to cell viability. A: MCF7 cells were treated for three hours with Aumitin in DMEM with or without glucose. B: MCF7 cells were treated for three hours with Rotenone in DMEM with or without glucose. C: MCF7 cells were treated for 24 hours with Aumitin in DMEM with or without glucose. D: MCF7 cells were treated for 24 hours with Rotenone in DMEM with or without glucose. Data is mean ±SD, n = 3, representative graphs shown.

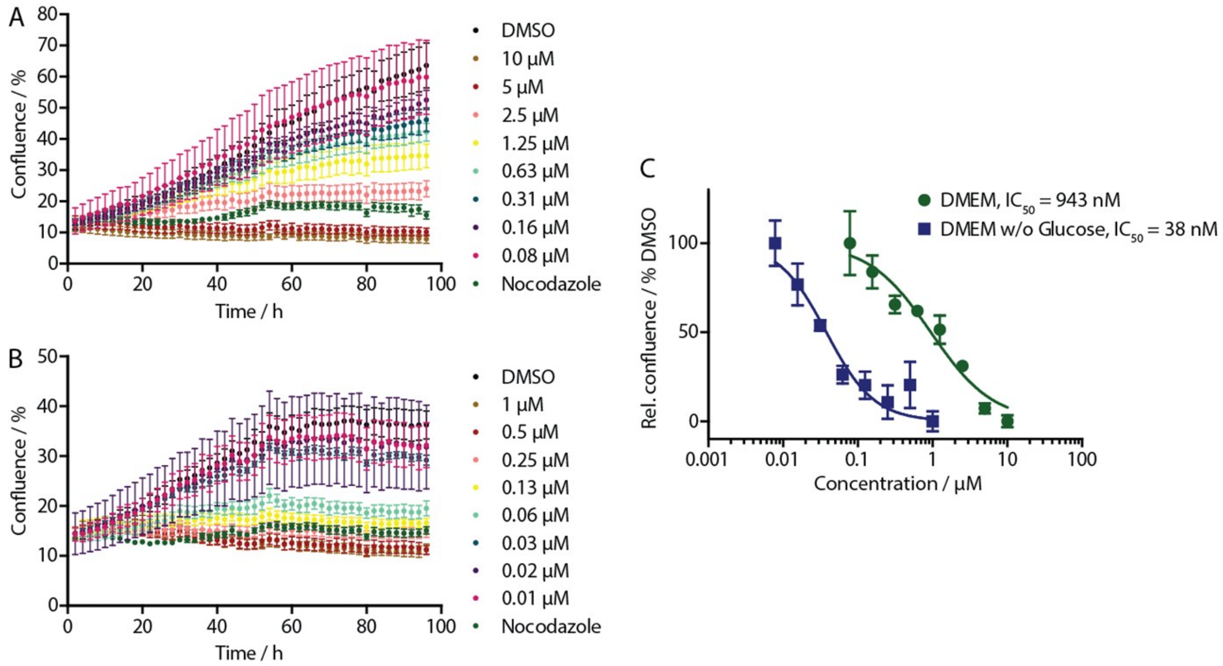

SI-Figure 10: Selective viability of MCF7 cells upon pharmacological mitochondrial complex I inhibition by means of Aumitin treatment. Confluence was assessed by phase contrast. The experiments were performed with an Incucyte Zoom instrument. **A:** MCF7 cells were cultured and incubated in DMEM when treated with Aumitin. **B:** MCF7 cells were cultured and incubated in DMEM without glucose when treated with Aumitin. **C:** Confluence (%) measured after 72 hours in A and B was normalized to the confluence for DMSO treated cells and plotted, in order to calculate IC_{50} values. Data is mean ± SD, n = 3, representative graphs shown.

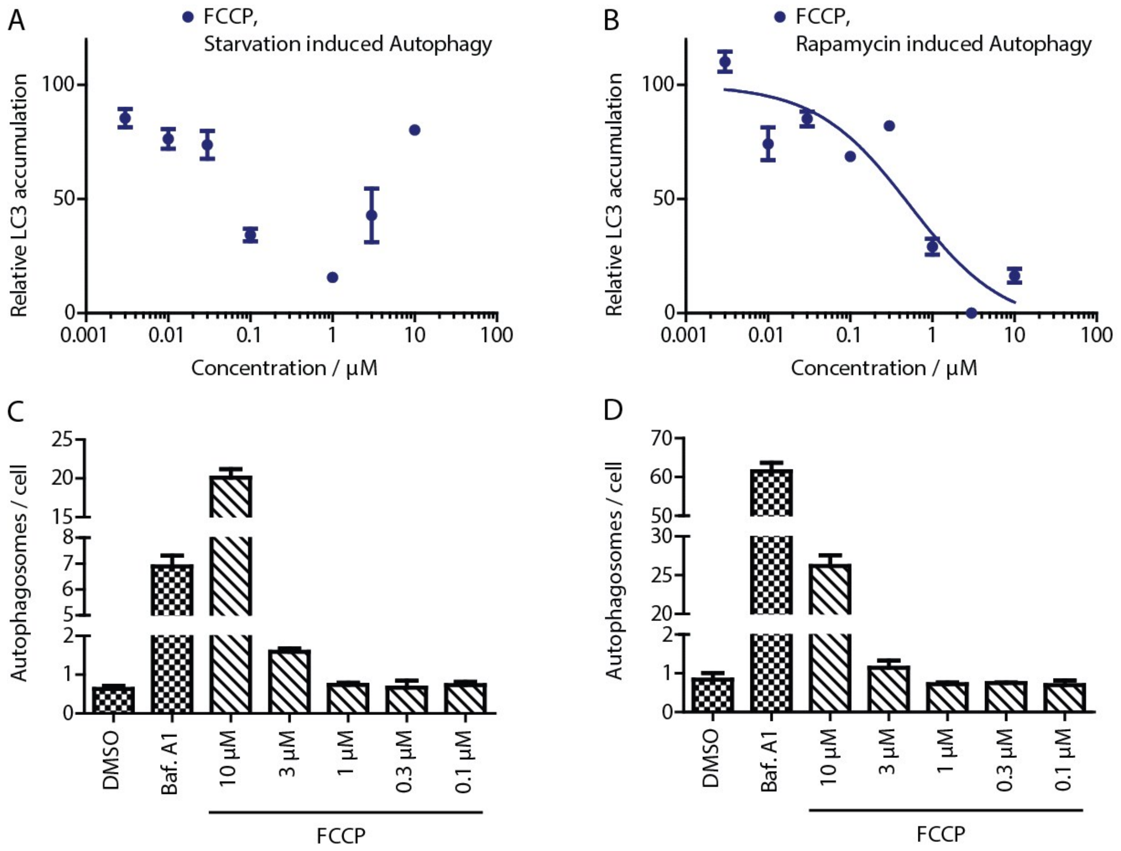

SI-Figure 11: The dose dependent effect of FCCP on autophagy. The effect on autophagy was assessed by means of measuring the number of eGFP-LC3 puncta in the aforementioned **MCF7-eGFP-LC3 cell line.** The concentration dependent effect of FCCP seems to vary within the two different autophagy inhibition assay, i.e. starvation or Rapamycin induced autophagy. Noteworthy is furthermore the observation that regardless of the assay format, i.e. under induced autophagy as well as under basal autophagy, only at higher concentrations, FCCP seems to increase the number of autophagosomes, whereas at lower concentrations it seems to inhibit autophagy. Many literature reports however use FCCP at high concentrations. **A** and **B:** Examination of FCCP's effect on the number of autophagosomes in the autophagy assay after three hours for the identification of autophagy inhibitors. **A:** Starvation by means of incubation of the cells in EBSS was used for the induction of autophagy. **B:** Rapamycin was used for the induction of autophagy. **C** and **D:** Effect of FCCP on the number of autophagosomes observed in **MCF7-eGFP-LC3 cells incubated under fed conditions by means of incubation in MEM. The number of autophagosomes observed for the DMSO treated sample is considered to be accounted for by basal autophagy. Bafilomycin (Baf. A1, 100 nM) was used as a positive control for an increased number of autophagosomes. C: The cells were incubated and treated with the respective compound for three hours. D: The cells were incubated and treated with the respective compound for 24 hours.** All data is mean ± SD, n = 3, representative experiments shown.

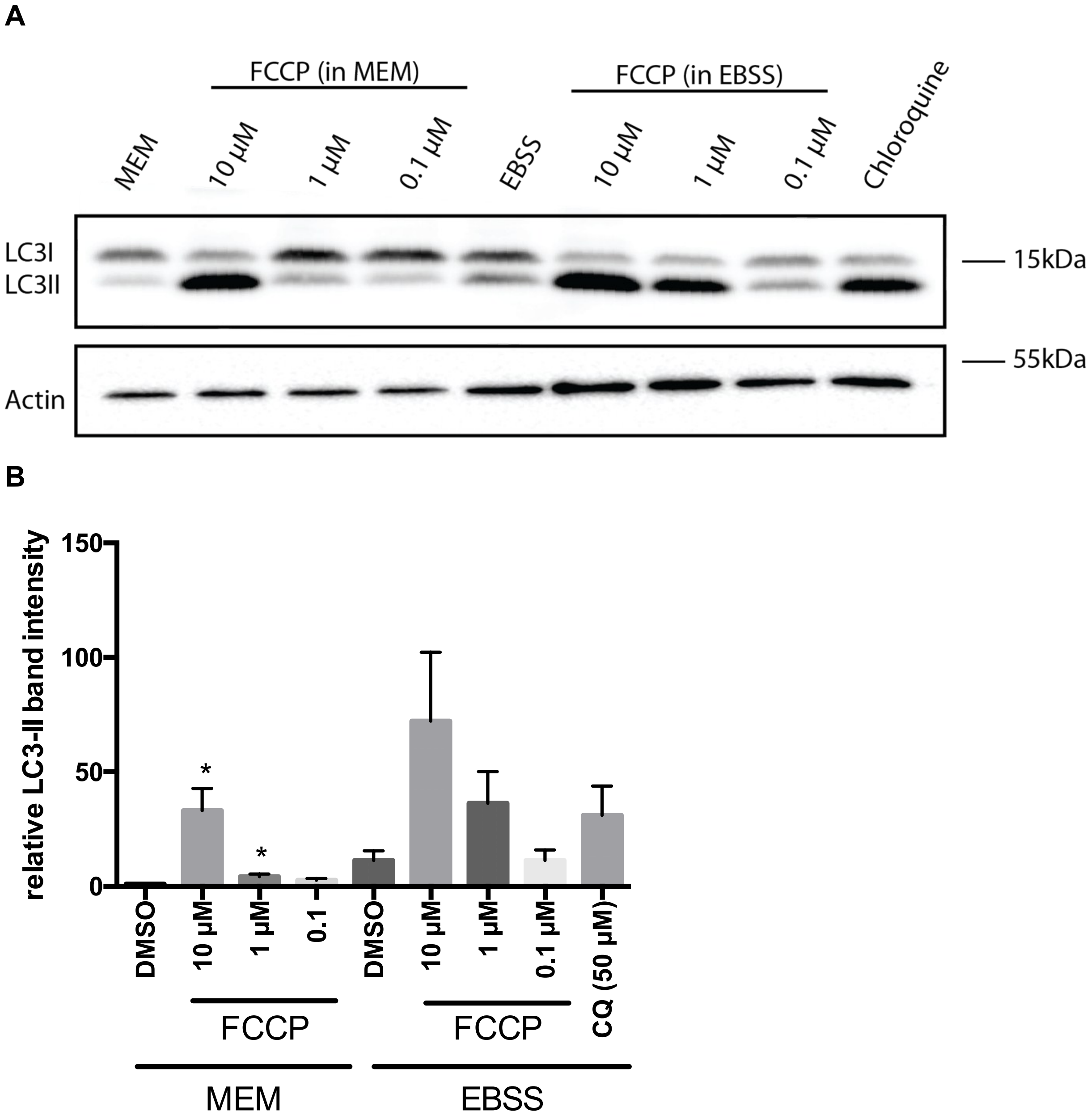

SI-Figure 12: Analysis of LC3 lipidation in response to FCCP in MCF7-eGFP-LC3 cells. **A:** representative Western blot. **B**: quantification of 3 independent experiments (n = 3). Band intensities were normalized to the MEM (DMSO) control. FCCP modulated LC3 lipidation in a dose-dependent fashion. p = 0.031 (10 µM FCCP vs DMSO in MEM) and p = 0.049 (1 µM FCCP vs DMSO in MEM) as assessed by the Students t-test. Chloroquine (CQ) was used at 50 µM.

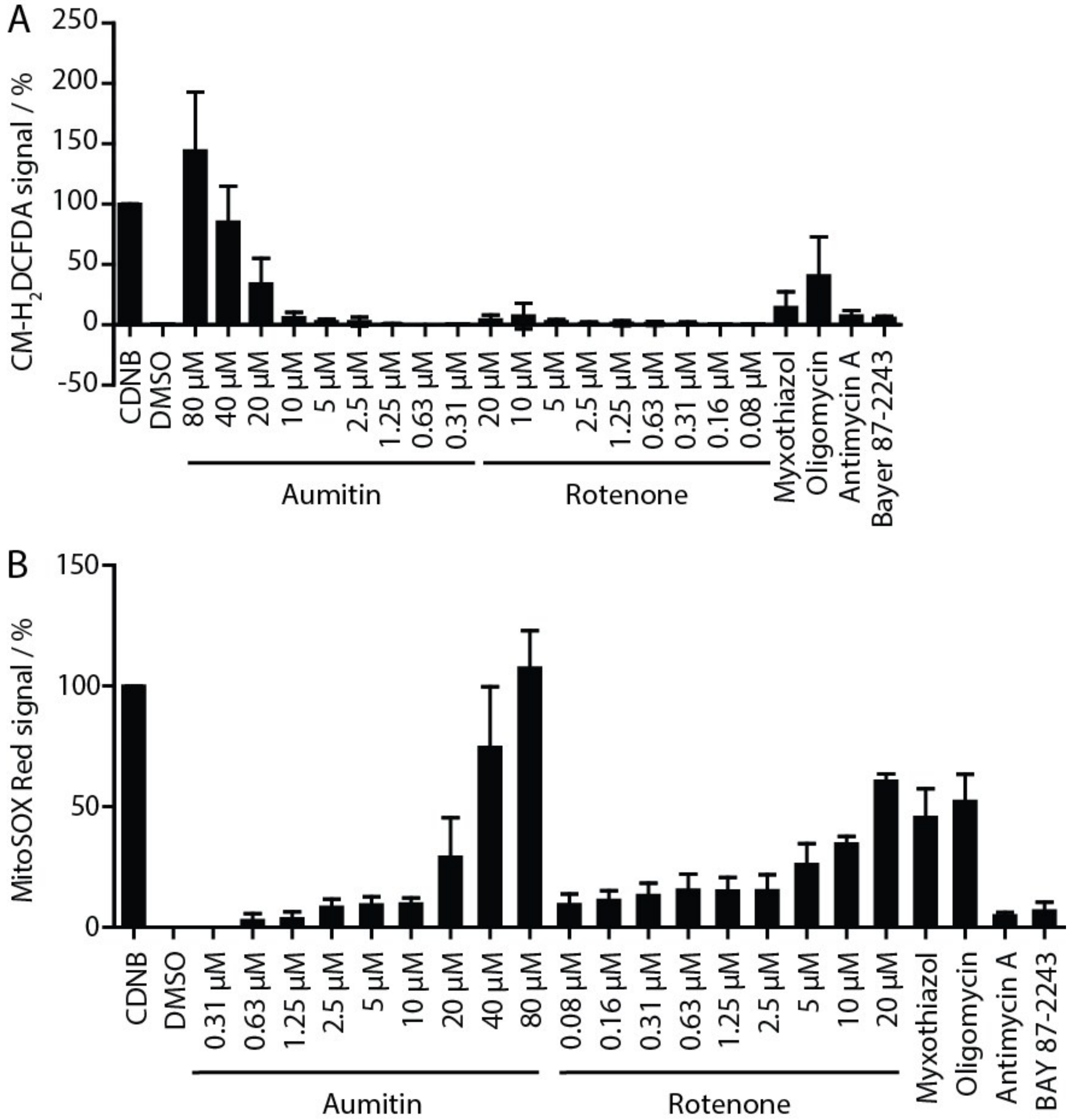

SI-Figure 13: Evaluation of the effect on ROS levels by mitochondrial respiration inhibitors. **A:** Aumitin and Rotenone generally induced the formation of ROS in HeLa cells dose-dependently after 1 h but only at higher concentrations to an extent comparable to the known ROS inducer dinitrochlorobenzene (CDNB, 10 µM). Myxothiazol, Oligomycin, Antimycin A and BAY87-2243 were used at a concentration of 10 µM. **B:** Aumitin and Rotenone dose-dependently induced the formation of ROS in mitochondria as examined by the MitoSOX Red dye after 1 h. Only at higher concentrations the induction of ROS was comparable to the effect of dinitrochlorobenzene (CDNB, 10 µM). Myxothiazol, Oligomycin, Antimycin A and BAY87-2243 were used at a concentration of 10 µM. All data is mean ± SD, n = 3. Data was normalized to cells treated with DMSO (= 0%) or 10 µM of CDNB (= 100%).

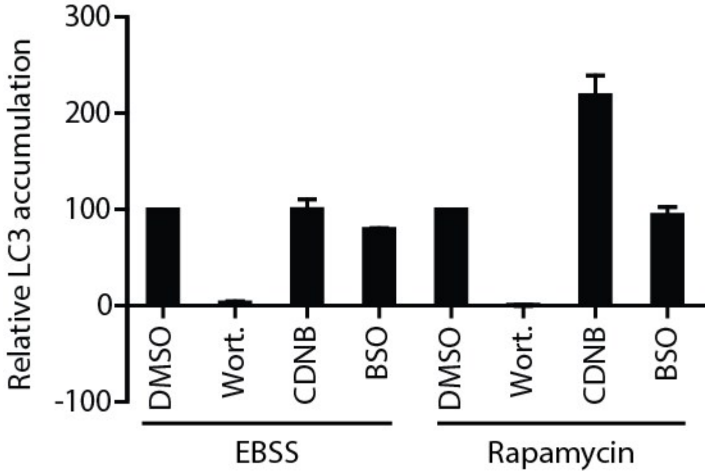

SI-Figure 14: Evaluation of the known ROS inducers L-Buthionine-sulfoximine (BSO) and dinitrochlorobenzene (CDNB) as autophagy inhibitors. BSO and CDNB did not inhibit starvation- or Rapamycin induced autophagy. BSO, CDNB and Wortmannin (Wort.) were used at a concentration of 10 µM. The relative LC3 accumulation was normalized to the value of DMSO treated cells for starvation- and Rapamycin induced autophagy respectively. Data is mean ± SD, n = 3, representative experiment shown.

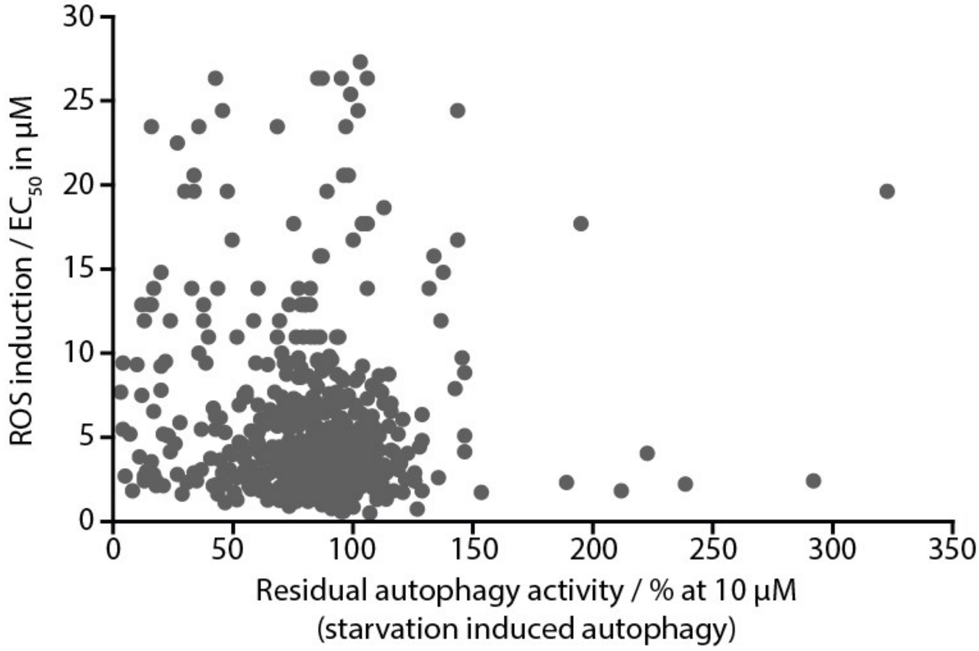

SI-Figure 15: Scatter plot for the correlation of ROS induction and autophagy inhibition. The EC_{50} values of 606 ROS inducers, identified in our in house screen (unpublished data), were plotted against the percentual inhibition of starvation induced autophagy at a concentration of 10 µM.

Methods and reagents

1.1.1 Antibodies for Western blotting

Target	Host	Cat#	Manufacturer	Dilution	Incubation Conditions
Anti-rabbit-HRP	goat	31460	Pierce	1:10,000	5% (w/v) milk in TBS-T
β-actin	rabbit	ab8227	Abcam	1:10,000	5% (w/v) milk in TBS-T
p62	rabbit	PM045	MBL international	1:10,000	5% (w/v) milk in TBS-T
LC3-B	rabbit	2775	Cell Signaling	1:1,000	5% (w/v) milk in TBS-T
VPS34	rabbit	3358	Cell Signaling	1:1,000	5% (w/v) BSA in TBS-T

1.2 Cell culture

All cell culture work involving living mammalian cells was performed with sterile equipment, media, and solutions in cell culture-approved benches. Waste was autoclaved for 15 min at 134 °C.
MCF7 cells stably transfected with eGFP-LC3 (MCF7-eGFP-LC3 cells) were cultured at 37 °C with 5% CO_2 using Eagle's MEM (Gibco cat# 21090-022) containing 10% (v/v) FBS (Invitrogen cat# 10500-084), 1 mM L-Glutamine (Invitrogen cat# 25030-081), 1 mM sodium pyruvate (PAN Biotech cat# P04-43100), 1% (v/v) NEAA (PAN Biotech cat# P08-32100), 0.01 mg/ml bovine insulin (Sigma Aldrich cat# I9278) and 200 µg/ml G418. Untransfected MCF7 cells were incubated in Eagle's MEM including the same additives as used for transfected cells except for G418.
HeLa cells were cultured in standard DMEM (PAN Biotech, cat# P04-03500) supplemented with 10% (v/v) FBS (Invitrogen cat# 10500-084), 1 mM sodium pyruvate (PAN Biotech, cat# P04-43100) and 1% (v/v) NEAA (PAN Biotech, cat# P08-32100) at 37 °C with 5% CO_2.

1.2.1 Thawing and Freezing Cryo-Conserved Cells

Thawing of frozen cells was performed by transferring the vial with the cells into a 37 °C warm water bath. The thawed cells were then diluted with 10 mL of the respective medium and the resulting suspension was transferred into a T-75 flask.

The cells were incubated as described above. On the next day the DMSO-containing medium was replaced by DMSO-free medium.
For cryo-conservation of mammalian cells, these were grown until reaching 80–95% confluence and then detached by trypsinization. The detached cells were suspended in the respective cell media to inactivate the trypsin, and the cell number was determined. The suspension was centrifuged (room temperature, 150 g, 5 min) and the medium replaced by an appropriate volume of DMSO-containing medium (5% v/v) to reach a final concentration of around 1×10^6 cells per cryo-vial). The vials were frozen in a cell freezing container (CoolCell® LX) at -80 °C overnight, to ensure a temperature decrease of 1 °C/min, and then stored in liquid nitrogen for long-term storage.

1.2.2 Passaging Mammalian Cells

MCF7, MCF7-eGFP-LC3 and HeLa cells were grown to a maximum confluence of 80–90%. The medium was removed, cells were washed with PBS (1X, 5 mL for 75 cm^2 (T-75), 10 mL for 175 cm^2 (T-175) tissue culture flasks) and then detached from the flasks by trypsinization (Trypsin/EDTA, 1 mL for T-75 flask, 3 mL for T-175 flask flasks, 6 min incubation at the conditions described above). After the incubation with trypsin, medium was added.

1.2.3 Determination of Cell Number

The concentration or number of cells were determined with the help of the Invitrogen™ Countess™ II Automated Cell Counter (Thermo Fisher Scientific, cat# AMQAX1000) together with Countess® Cell Counting Chamber Slides (Thermo Fisher Scientific, cat# C10228) according to the manufacturer's instructions.

1.2.4 Check for Mycoplasma Contaminations in Cultured Cells

All used cells were checked for mycoplasma contamination using the MycoAlert™ Mycoplasma Detection Kit (Lonza) (cat# LT07) according to manufacturer's instructions.

1.2.5 Cell Treatment with Small Molecules

If not stated otherwise all small molecules were dissolved in DMSO (Bioreagent, Sigma Aldrich, cat# D8418). Cells were seeded into vessels in a cell number that allowed 75–90% confluence at the end of the experiment. Cells were incubated for 12-24 hours as described above and then treated with compounds, which were pre-diluted in the given medium. The final DMSO concentration did not exceed 0.5% (v/v).

1.3 Autophagy Assay

1.3.1 High-content screening for autophagy inhibitors

The phenotypic autophagy screen utilizes a MCF7 cell line stably expressing eGFP-tagged LC3 protein. 4,000 cells per well were seeded in 25 µl medium in a 384-well Greiner µclear plate (cat# 781080, lid cat# 656191) and incubated (37 °C, 5% CO_2) overnight. Cells were then washed by a plate washer (Biotek, ELx405) three times with PBS followed by a final aspiration of the washing buffer. The addition of 25 nl of compound solution (10 mM stock solution in DMSO) was then carried out with an echo dispenser (Labcyte, Echo 520 dispenser). Addition of medium to induce autophagy was carried out with a Multidrop Combi (Thermo Scientific). 25 µl EBSS (Sigma Aldrich, cat# E3024-500ml) containing 50 µM Chloroquine (Sigma Aldrich, cat# C6628-25g) was used for starvation-induced autophagy and 25 µl medium containing 50 µM Chloroquine and 100 nM Rapamycin (Biomol, cat# Cay13346) was used for rapamycin-induced autophagy screening. After incubation (37 °C, 5% CO_2) for three hours cells were fixed by addition of 25 µl 1:4 formaldehyde in PBS + 1:500 Hoechst (stock: 1 mg/ml, Sigma Aldrich cat# B2261-25mg) and incubation for 20 min at room temperature. Cells were then washed three times with 1X PBS. Four images per well were taken with ImageXpress Micro XL (Molecular Devices) at 20x. Automated image analysis was performed using the granularity setting of MetaXpress Software (Molecular Devices). The most significant analysis parameter was granule area; with resulting signal-to-background ratios around 40 and Z' values around 0.7. The screening of 160,000 compounds yielded ca. 7,000 hit compounds which suppressed the autophagosome content by more than 50%.

1.3.2 Low-throughput Screening for Autophagy Inhibitors

The low-throughput version utilizes the same MCF7-eGFP-LC3 cell line as the above described high-throughput version (1.3.1). 10,000 cells per well were seeded in 100 µL medium in 96-well Corning® CellBIND® plates (cat# 3340) and incubated overnight. Cells were washed with 50 µL 1X PBS followed by an aspiration of the washing buffer. Autophagy was induced by addition of 100 µL EBSS (100 µL) containing 50 µM Chloroquine prior to compound addition (20 µL, diluted in EBSS). After incubation (37 °C, 5% CO_2) for 3 h cells were washed with PBS and subsequently fixed by addition of 25 µL 4% formaldehyde in PBS + 1:500 Hoechst (stock: 1 mg/ml, Sigma Aldrich, cat# B2261-25mg) for 20 min at room temperature. Cells were then washed twice with 100 µL and stored in 100 µL PBS. Four images per well were taken with an automated AxioVert 200M fluorescence microscope (Zeiss) at 20X. Automated image analysis was performed using a modified configuration of the granularity assay (approx. granule min. and max. width: 0.4 and 5 µm resp.; approx. nuclei min. and max. width: 6 and 20 µm resp.; intensity of granules and nuclei above background: 300 and 40 gray levels resp.) of MetaMorph Cell Analysis Software (Molecular Devices). Inhibition of autophagy was quantified by calculating the number of granules per nucleus, normalized to the DMSO-treated controls.

1.4 Protein Concentration Determination

Routine determination of protein concentration was performed using the Bradford method with the Bradford reagent (Bio-Rad laboratories) in the linear range of a BSA control (0.3 –10 mg/mL). The absorbance was measured at 595 nm using a BioPhotometer (Eppendorf).

For a large number of samples or low protein concentrations, a DC protein assay (Bio-Rad laboratories, cat# 500-0116) was performed in 96-well plates according to the manufacturer's instructions. The absorbance was measured at 650–750 nm using a Tecan microplate reader (Tecan Infinite M200) and the concentration calculated using a BSA standard curve.

1.5 Detection of p62 conversion and LC3-I to LC3-II ratios by means of immunoblotting

200,000 MCF7-eGFP-LC3 cells in 2 ml media were seeded in 6-well plates and incubated (37 °C, 5% CO_2) overnight. The media was removed and the cells were washed with PBS (one time), before adding test compounds at the required concentrations in EBSS (2 mL). Cells were incubated (37 °C, 5% CO_2) for 3 h before removing the media, washing with PBS (one time), and lysing in 100 µL SDS loading buffer without bromophenol blue and DTT. Protein concentrations were determined using the DC Assay (Bio-rad) according to the manufacturer's instructions. . After addition of bromophenol blue (1:1000 from a 1% bromophenol blue stock solution) and DTT (final concentration of 50 mM from a 1 M Stock solution) the samples were boiled at 95 °C for 5 min. SDS-PAGE was carried out using self-cast 15% polyacrylamide gels run at a constant voltage of 80 V for 15 min followed by 120 V for approximately 2 h. Semi-dry transfer onto a PVDF membrane (Immobillion-FL, prod# IPFL00010) was performed at 25 V for 45 min. Membranes were blocked in 5% (w/v) powdered milk in TBS-T (blocking buffer) for 1 h at room temperature. The membrane was incubated with the primary antibody in blocking buffer overnight at 4 °C. After washing with TBS-T (3 x 5 min) the membrane was incubated with a HRP-coupled secondary antibody in blocking buffer for one hour at room temperature. Signals were visualized using the SuperSignal West Pico Chemiluminescent Substrate or the SuperSignal West Femto Maximum Sensitivity Substrate (Thermo Fischer) and an Odyssey® FC Imaging System (LICOR). Work was conducted as proposed by the guidelines for the use and interpretation of assays for monitoring autophagy.[5]

1.6 Detection of apoptosis by fluorescence live cell imaging

This method consists of cell treatment with IncuCyte™ Caspase-3/7 Apoptosis Assay Reagent (Essen Bioscience, cat# 4440), a fluorescently-tagged peptide that is recognized and cleaved by the apoptotic caspases 3 and 7. This triggers the release of a DNA-intercalating fluorophore which stains nuclei. Using automated image analysis, fluorescent nuclei can be quantified and are directly proportional to the number of apoptotic cells.

30,000 (for fed conditions) or 100,000 (for starved conditions) MCF7 cells in 100 µL medium were seeded in a clear flat-bottomed 96-well plate and incubated overnight. The medium was removed carefully, and cells were washed with PBS (50 µL), and 100 µL of medium (DMEM for fed, EBSS for starved conditions) containing 1.2% (v/v) IncuCyte™ Kinetic Caspase-3/7 Apoptosis Assay Reagent. Test compounds in a DMSO stock solution were prediluted in the appropriate medium (MEM or EBSS) at a concentration of 6x the desired final concentration, while keeping the DMSO content constant. Of this solution, 20 µL were added to the cells, and the cells were incubated at 37 °C and 5% CO_2 between 24 and 72 h in the Incucyte™ Zoom. Images were acquired at 10X magnification in both the phase and green channel every hour. Images were analyzed using automated image analysis with the Incucyte™ Zoom Control Software. The key parameter was green fluorescence confluence as a percentage of phase contrast confluence (%). Data was expressed as a percentage of the DMSO-treated sample.

1.7 Viability assay, WST-1 assay

4,000 cells in 100 µl MEM were seeded in a clear flat-bottomed 96-well plate and incubated overnight. For starved conditions, 7000 cells in 100 µL EBSS were used. The medium was removed gently, and cells were washed with PBS (100 µL). The PBS was replaced with 100 µL of media. The test compounds were added at a concentration of 6X the desired final concentration in 20 µl of the appropriate medium. Cells were incubated at 37 °C for 48 h. At this point 10 µl of WST-1 reagent (Roche) were added to each well. The absorbance was measured with a Beckman DTX-880 (Beckman Coulter, Germany) plate reader at 450/690 nm. Absorbance measurements were performed after 20, 30, 40, 50, 60 min of addition. Good measurements are considered when absorbance of the negative control is around 1.00. Growth inhibition values (GI_{50}) were calculated using Graphpad Prism using DMSO (negative) and Nocodazole (10 µM, positive) as controls.

1.8 CellTiter-Glo® Luminescent Cell Viability Assay

The assay is a Luciferin based measurement of ATP-levels. The ATP-levels are considered to be proportional to the amount of viable and metabolically active cells.

300 MCF7 cells were seeded in 25 µL MEM (without phenol red and G418) per well in a 384-well plate (Greiner, cat# 781080) and incubated for 24 hours (37 °C, 5% CO_2). Cells were then washed by a plate washer (Biotek, ELx405) three times with PBS followed by a final aspiration of the washing buffer. The addition of 25 nL of compound solution (10 mM stock solution in DMSO, highest final compound concentration was 10 µM) was then carried out with an echo dispenser (Labcyte, Echo 520 dispenser). Addition of 25 µL medium (MEM (Gibco, cat# 21090-022), EBSS (Sigma Aldrich, cat# E3024-500ml) or DMEM w/o glucose (PAN Biotech, cat# P04-01548S1)) was carried out with a Multidrop Combi (Thermo Scientific). The plate was then incubated as described above for 3 or 24 hours and afterwards 15 µL CellTiter-Glo® Reagent (Promega, cat# G7570) were added into each well. The chemiluminescent signal was read out by a Spectramax Paradigm (Molecular Devices) with its high sensitivity luminescence cassette.

1.9 Determination of Induction of Reactive Oxygen Species (ROS)

1.9.1 Cell Culture

HeLa cells and U2OS cells were cultured in standard DMEM (PAN Biotech, cat# P04-03500) supplemented with 10% (v/v) FBS (Invitrogen cat# 10500-084), 1 mM sodium pyruvate (PAN Biotech, cat# P04-43100) and 1% (v/v) NEAA (PAN Biotech, cat# P08-32100) at 37 °C with 5% CO2.

1.9.2 Measurement of Cellular Reactive Oxygen Species (ROS) Levels

Cellular ROS levels were determined using the general ROS indicator CM-H_2DCFDA (ThermoFisher Scientific, cat# C6827). HeLa cells were seeded into black 96 well plates with clear flat bottom (Corning) and incubated at 37 °C, 5% CO2 overnight. Test compounds were added followed by 60 min of incubation. The medium was exchanged for DMEM (without additives) containing 5 µM CM-H_2DCFDA and 5 µg/µL Hoechst-33342 (ThermoFisher Scientific, cat# H3570) and cells were incubated for 30 min at 37°C. Cells were fixed with 0.5% paraformaldehyde in phosphate-buffered saline (PBS) for 10 min at room temperature. Afterwards, cells were washed three

times with PBS. Cells were imaged at 10x magnification using an Axiovert 200M automated fluorescence microscope (Carl Zeiss, Germany). CM-H_2DCFDA-stained area per cell was quantified using MetaMorph 7.7.8.0 (Visitron, Germany). Data was normalized to cells treated with DMSO (= 0%) or 10 µM of CDNB (= 100%). Mean values of three individual experiments are shown.

1.9.3 Measurement of Mitochondrial Reactive Oxygen Species (ROS) Levels

For the determination of mitochondrial superoxide levels the indicator MitoSOX Red (ThermoFisher Scientific, cat# M36008) was used. HeLa cells were seeded and incubated, as described above. Cells were incubated in DMEM (without additives) containing 5 µM MitoSOX Red and 5 µg/µL Hoechst-33342 for 30 min at 37°C, 5% CO2. The supernatant was removed and test compound-containing medium was added, followed by 60 min of incubation at 37°C, 5% CO_2. Cells were fixed, washed and imaged as described above. MitoSOX Red-stained area was quantified using CellProfiler.2.2.0 Data was normalized as described above. Mean values of three individual experiments are shown.

1.10 Detection of Mitochondrial Respiration Inhibition

1.10.1 Cell culture

The human cervix epidermoid carcinoma cell line HeLa and the human breast adenocarcinoma cell line MCF7 were obtained from RIKEN Cell Bank and verified to be free of mycoplasma contamination by Hoechst staining. Both cell lines were cultured at 37 °C in DMEM (Thermo Fisher Scientific), supplemented with 10% (v/v) FBS (Sigma-Aldrich).

1.10.2 Measurement of Mitochondrial Respiration

The oxygen consumption rate (OCR) and the extracellular acidification rate (ECAR) were measured by the Seahorse XFe96 analyzer (Agilent). HeLa cells (1×10^4 cells/well) or MCF7 cells (0.8 1×10^4 cells/well) plated in XFe96 microplates (Agilent) were preincubated in DMEM-based assay medium (Agilent) supplemented with

25 mM glucose, 2 mM GlutaMAX (Thermo Fisher Scientific), and 1 mM sodium pyruvate (Sigma-Aldrich) for 1 h at 37 °C in a CO_2 free incubator. After the baseline measurements of OCR/ECAR, a test sample was injected from port A. The changes in OCR/ECAR values were recorded in triplicate ten times every 6 minutes, followed by the measurements of XF Cell Mito Stress Test (Agilent), i.e. sequential injections of oligomycin A (1 µM) from port B, FCCP (125 nM) from port C, and antimycin A (1 µM) plus rotenone (1 µM) from port D.

1.10.3 Semi-intact Assay for Mitochondrial Respiration

The semi-intact assay for mitochondrial respiration was carried out by the established method[6] with some modifications in the concentrations of digitonin and substrates. HeLa cells (1×10^4 cells/well) were preincubated in MAS buffer (220 mM mannitol, 70 mM sucrose, 10 mM KH_2PO_4, 5 mM $MgCl_2$, 2 mM HEPES-KOH and 1 mM EGTA; pH 7.2) for 15 min at 37 °C in a CO_2 free incubator. After three baseline measurements of OCR, digitonin (20 µg/ml), sodium succinate (10 mM), Rotenone (1 µM), ADP (1 mM), and test samples were injected from port A to start the reaction, followed by sequential treatments of Oligomycin A (1 µM) from port B and Antimycin A (1 µM) or sodium azide (20 mM) from port C. Succinate plus Rotenone were replaced with malate (1 mM) plus pyruvate (10 mM) for the assessment of complex I, duroquinol (0.5 mM) for complex III, or TMPD (0.5 mM) plus sodium ascorbate (2 mM) for complex IV.

1.10.3.1 NADH-CoQ reductase activity test

The enzymatic activity of complex I was determined with a MitoCheck complex I assay kit (Cayman, cat# 700930) according to the manufacturer’s instructions.

1.11 Confocal live cell imaging of mCherry-eGFP-LC3/MCF7 cells

400,000 stable MCF7-mCherry-eGFP-LC3 cells were seeded in glass bottom dishes (MatTek, Ashland) and incubated overnight (37 °C, 5% CO_2). On the next day the medium was removed and replaced with 2 mL of the indicated treatment medium containing the appropriate compounds. After incubation (37 °C, 5% CO_2) for three hours live cell imaging was performed in MEM without phenol red (Invitrogen, cat#

51200-046) or EBSS (Sigma Aldrich, cat# E3024-500ml) by using an inverted confocal microscope Leica SP5 AOBS equipped with a 63×/1.4 HCX Plan Apo oil imMersion lens and a temperature-controlled hood at 37°C and 5% CO_2. The ImageJ software was used to quantify Green and Red Puncta Colocalization macro to analyze mCherry-eGFP-LC3 puncta (Ruben K. Dagda, University of Nevada School of Medicine, Pharmacology Department; Daniel Shiwarski, Carneggie Mellon University; Charleen T Chu, University of Pittsburgh).[7]

1.12 Detection of biochemical kinase inhibition

The detection of biochemical inhibition of PI4KB and PIK3C2G as well as the full panel including overall 419 kinases was carried out by Life Technologies (Thermo Fisher Scientific) using the Select Screen® kinase profiling service. Procedures and conditions can be found online. Measurements were performed in duplicate according to the supplier's standards.

1.13 Synthesis of Aumitin

Techniques: All reactions involving air or moisture sensitive reagents or intermediates were carried out following standard Schlenk line technique under an argon atmosphere and all the glassware was dried by heat gun under high vacuum prior to use. Concentration of the reaction mixture was performed under reduced pressure at 40°C at the appropriate pressure. Purified compounds were further dried under high vacuum.

Microwave: Reactions performed in the microwave were carried out in the CEM Focused Microwave™ Synthesis System, Discover® SP. The reaction mixture was stirred for one minute before the start of the irradiation, in order to ensure a homogenous mixture. Pressure stable vessels with air vent caps were used. If not stated otherwise 200 Watt were applied to the vessel / the reaction mixture, in order to heat it.

Solvents and reagents: Dry solvents were received from Acros in anhydrous quality and used without any further purification. Acetone was dried by stirring it over anhydrous potassium carbonate or magnesium sulfate for 1 hour. All other solvents or reagents were purified according to standard procedures or were used as received from Sigma Aldrich, Alfa Aesar, Acros, Activate Scientific, Matrix Scientific, Combi Blocks, Fisher Scientific, Merck and TCI. Milli-Q grade water was used for all experiments.

TLC: TLC was performed using pre-coated Merck silica gel 60 F254 glass plates, detection of compounds were performed by UV254 light and/or dipping into a solution of KMnO4 (1.5 g in 400 mL H_2O, 5 g $NaHCO_3$) followed by heating with a heat gun.

Column chromatography: Column chromatography was performed using silica gel from Acros Organics (40 – 65 µm, 230 – 400 mesh). The overpressure was between 0.2 and 0.5 bar. Eluent mixtures are shown in parentheses and stated in volume percent for the polar solvent (X% V_1 / V_2) or in volume percent plus total volume percent (X% V_1 / V_2 + Y% V_3). Used eluents are stated in each section.

^{1}H-NMR and ^{13}C-NMR: Were recorded on devices from Bruker (AMX 400 MHz, AMX 500 MHz and AMX 600 MHz) and on a device from Varian (Mercury 400 MHz), using $CDCl_3$ or DMSO-d_6, CD_2Cl_2, $(CD_3)_2CO$, CD_3CN or CD_3OD as solvent. Data are reported in the following order: chemical shift (δ) values are reported in ppm with the solvent resonance as internal standard ($CDCl_3$: δ = 7.26 ppm for ^{1}H, δ = 77.16 ppm for ^{13}C), (DMSO-d_6: δ = 2.50 ppm for ^{1}H, δ = 39.52 for ^{13}C), (CD_2Cl_2: δ = 5.32 ppm for ^{1}H, δ = 53.84 for ^{13}C), ($(CD_3)_2CO$: δ = 2.05 ppm for ^{1}H, δ = 29.84 ppm for ^{13}C), (CD_3CN: δ = 1.94 ppm for ^{1}H, δ = 1.32 ppm for ^{13}C), (CD_3OD: δ = 3.31 ppm for ^{1}H, δ = 49.00 ppm for ^{13}C) multiplicities are indicated bs (broadened singlet), s (singlet), d (doublet), t (triplet), q (quartet), m (multiplet), dd (doublet of doublet), dt (doublet of triplet); coupling constants (*J*) are given in Hertz (Hz). Unless stated otherwise all NMRs were measured at room temperature. Where possible, structural assignments were attempted using standard 2-D NMR techniques (gCOSY, gHSQC, gHMBC).

Analytical HPLC-MS: Data were recorded on one of the following three systems.
1: HPLC system (Ultimate 3000 by Thermo Fisher) with a *Nucleodur* EC 125/3 C4 or a *Nucleodur* EC 50/3 C18 reverse column (Macherey & Nagel) coupled to an ESI spectrometer (LCQ Fleet, Thermo Fisher).
2: HPLC system (Agilent Series 1100 by Agilent Technologies). A CC 125/4 C4 column (Nucleodur) or a CC 125/4 C18-column (Macherey & Nagel) coupled to an ESI spectrometer (LCQ Advantage MAX, Thermo Fisher).
3: uHPLC System (1290 Infinity by Agilent Technologies) with a C18 column (Zorbax Eclipse C18 Rapid Resolution, 2.1*50mm*1.8µm) coupled to an ESI spectrometer (6150 Agilent Technologies).

HRMS (ESI): Spectra were recorded on a LTQ Orbitrap (Thermo Fisher) mass spectrometer coupled to an Accela HPLC-System (HPLC column: Hypersyl GOLD, 50 mm x 1 mm, particle size 1.9 µm), ionization method: electron spray ionization.

Synthesis of Aumitin

2-chloro-6-methyl-*N*-phenylpyrimidin-4-amine

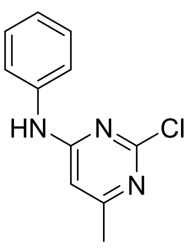

2,4-dichloro-6-methylpyrimidine (8.00 g, 49.1 mmol), aniline (5.60 mL, 61.4 mmol) and DIPEA (10.69 mL, 61.4 mmol) were dissolved in cold *n*-butanol (80 mL) and slowly brought to rt over 3 days. Subsequently, 100 mL of EtOAc and 40 mL of water were added to the reaction solution. The aqueous layer was extracted three times with 40 mL of EtOAc and the combined organic layers were dried over anhydrous magnesium sulfate and then concentrated *in vacuo*. The crude was purified by column chromatography on silica gel (0 – 50% EtOAc/cyclohexane). The product (4.50 g, 20.5 mmol, 42%) was obtained as an off-white solid.

^{1}H NMR (600 MHz, DMSO-d_6) δ = 9.89 (s, 1H), 7.55 (d, *J* = 7.8 Hz, 2H), 7.36 (t, *J* = 7.9 Hz, 2H), 7.08 (t, *J* = 7.4 Hz, 1H), 6.58 (s, 1H), 2.27 (s, 3H). ^{13}C NMR (151 MHz, DMSO) δ = 167.24, 161.89, 159.02, 138.84, 128.98, 123.40, 120.53, 103.44, 23.20.

ESI-MS: m/z = 220.23, 222.11 $[M+H]^+$.

HRMS (ESI): calculated for $C_{11}H_{11}N_3{}^{35}Cl$: m/z = 220.06360 $[M+H]^+$,
found: m/z = 220.06424 $[M+H]^+$.
calculated for $C_{11}H_{11}N_3{}^{37}Cl$: m/z = 222.06065 $[M+H]^+$,
found: m/z = 222.06086 $[M+H]^+$.

***N*2-(4-aminophenyl)-6-methyl-*N*4-phenylpyrimidine-2,4-diamine**

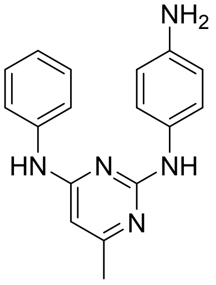

2-chloro-6-methyl-*N*-phenylpyrimidin-4-amine (1.00 g, 4.55 mmol) and *p*-phenylenediamine (492 mg, 4.55 mmol) were dissolved in 15 mL EtOH and heated to 50 °C under microwave irradiation for 1 h. Afterwards the ethanol was removed *in vacuo* and the product used without further purification.

^{1}H NMR (600 MHz, CD_3CN) δ = 7.62 (bs, 2H), 7.52 (d, *J* = 7.8 Hz, 2H), 7.27 – 7.19 (m, 4H), 6.96 (t, *J* = 7.4 Hz, 1H), 6.58 – 6.54 (m, 2H), 5.93 (s, 1H), 3.77 (bs, 2H), 2.13 (s, 3H). ^{13}C NMR (151 MHz, CD_3CN) δ = 167.06, 162.47, 161.52, 144.36, 141.00, 131.77, 129.64, 123.67, 123.42, 121.43, 115.55, 96.57, 24.05.

ESI-MS: m/z = 292.30 $[M+H]^+$.

HRMS (ESI): calculated for $C_{17}H_{18}N_5$: m/z = 292.15567 $[M+H]^+$,
found: m/z = 292.15597 $[M+H]^+$.

2-chloro-*N*-(4-((4-methyl-6-(phenylamino)pyrimidin-2-yl)amino)phenyl)benz-amide (Aumitin, 1)

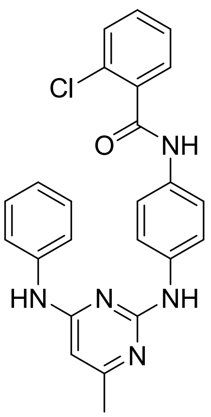

*N*2-(4-aminophenyl)-6-methyl-*N*4-phenylpyrimidines-2,4-diamine (54 mg, 185 µmol), 2-chlorobenzoic acid (29 mg, 185 µmol), DIPEA (32 µL, 185 µmol) and Pybop (96 mg, 185 µmol) were dissolved in 2 mL DMF at 0 °C. The reaction mixture was brought to room temperature overnight. The reaction was then quenched by the addition of 10 mL of water and 15 mL of EtOAc. The organic layer was washed twice with 4 mL of HCl (aq., 1M), twice with 4 mL of sodium carbonate solution (saturated, aq.) and eventually dried over anhydrous magnesium sulfate and concentrated *in vacuo*. The product (42 mg, 77 µmol, 42%) was isolated by chromatography on silica gel (0-50% EtOAc/cyclohexane)

Rf: 0.13 (50% EtOAc/cyclohexane)

^{1}H NMR (600 MHz, CD_3CN) δ = 8.64 (s, 1H), 7.92 (s, 1H), 7.65 (d, *J* = 8.9 Hz, 2H), 7.61 – 7.55 (m, 5H), 7.51 (dd, *J* = 8.0, 0.9 Hz, 1H), 7.46 (td, *J* = 7.7, 1.7 Hz, 1H), 7.42 (td, *J* = 7.4, 1.2 Hz, 1H), 7.34 (t, *J* = 7.9 Hz, 2H), 7.10 (t, *J* = 7.4 Hz, 1H), 6.08 (s, 1H), 2.26 (s, 3H). ^{13}C NMR (151 MHz, CD_3CN) δ = 165.93, 162.69, 158.93, 140.07, 137.64, 137.15, 134.37, 132.18, 131.46, 130.84, 129.98, 129.82, 128.15, 124.57, 122.29, 121.72, 121.33, 97.56, 22.65.

ESI-MS: m/z = 430.45, 432.38 [M+H]$^+$.

HRMS (ESI): calculated for $C_{24}H_{21}N_5{}^{35}Cl$: m/z = 430.14291 [M+H]$^+$,
found: m/z = 430.14394 [M+H]$^+$.
calculated for $C_{24}H_{21}N_5{}^{37}Cl$: m/z = 432.13996 [M+H]$^+$,
found: m/z = 432.14105 [M+H]$^+$.

Synthesis and origin of literature reported kinase inhibitors

PI4KB inhibitor 1

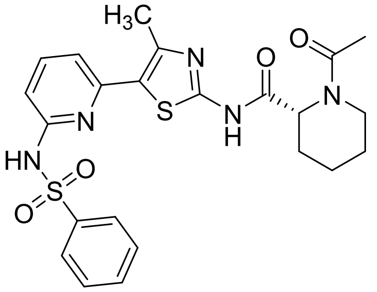

The thiazole based PI4KB inhibitor was a kind donation by Astra Zeneca.[1]

PI4KB inhibitor 2

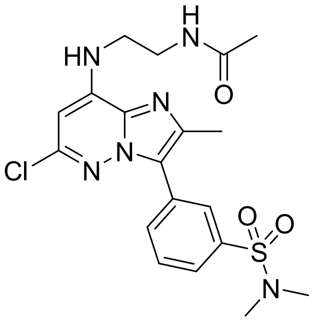

The imidazo[1,2-b]pyridazine based PI4KB inhibitor was synthesized as described in the literature.[2]

PIK3C2G inhibitors

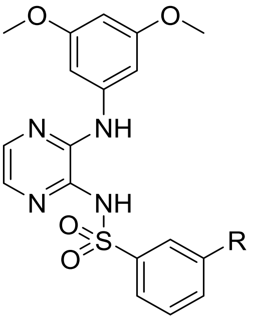

All PIK3C2G inhibitors were synthesized as described in the literature.[3]

References

1 M. J. Waring, D. M. Andrews, P. F. Faulder, V. Flemington, J. C. McKelvie, S. Maman, M. Preston, P. Raubo, G. R. Robb, K. Roberts, R. Rowlinson, J. M. Smith, M. E. Swarbrick, I. Treinies, J. J. G. Winter and R. J. Wood, *Chem. Commun.*, 2014, **50**, 5388.

2 I. Mejdrová, D. Chalupská, M. Kögler, M. Šála, P. Plačková, A. Baumlová, H. Hřebabecký, E. Procházková, M. Dejmek, R. Guillon, D. Strunin, J. Weber, G. Lee, G. Birkus, H. Mertlíková-Kaiserová, E. Boura and R. Nencka, *J. Med. Chem.*, 2015, **58**, 3767.

3 A. Freitag, P. Prajwal, A. Shymanets, C. Harteneck, B. Nürnberg, C. Schächtele, M. Kubbutat, F. Totzke and S. A. Laufer, *J. Med. Chem.*, 2015, **58**, 212.

4 S. J. Mountford, Z. Zheng, K. Sundaram, I. G. Jennings, J. R. Hamilton and P. E. Thompson, *ACS Med. Chem. Lett.*, 2015, **6**, 3.

5 D. J. Klionsky *et al.*, *Autophagy*, 2016, **12**, 1.

6 J. K. Salabei, A. A. Gibb and B. G. Hill, *Nat. protoc.*, 2014, **9**, 421.

7 O. Pampliega, I. Orhon, B. Patel, S. Sridhar, A. Díaz-Carretero, I. Beau, P. Codogno, B. H. Satir, P. Satir and A. M. Cuervo, *Nature*, 2013, **502**, 194.
